# Low‐glucose‐sensitive TRPC6 dysfunction drives hypoglycemia‐induced cognitive impairment in diabetes

**DOI:** 10.1002/ctm2.205

**Published:** 2020-10-18

**Authors:** Chengkang He, Peng Gao, Yuanting Cui, Qiang Li, Yingsha Li, Zongshi Lu, Huan Ma, Yu Zhao, Li Li, Fang Sun, Xiaowei Chen, Hongbo Jia, Daoyan Liu, Gangyi Yang, Hongting Zheng, Zhiming Zhu

**Affiliations:** ^1^ Department of Hypertension and Endocrinology, Center for Hypertension and Metabolic Diseases, Daping Hospital Chongqing Institute of Hypertension Army Medical University Chongqing China; ^2^ Brain Research Center Army Medical University Chongqing China; ^3^ Suzhou Institute of Biomedical Engineering and Technology Chinese Academy of Sciences Suzhou China; ^4^ Endocrine Department Second Affiliated Hospital of Chongqing Medical University Chongqing China; ^5^ Department of Endocrinology Translational Research Key Laboratory for Diabetes, Xinqiao Hospital Army Medical University Chongqing China

**Keywords:** cognition impairment, diabetes, recurrent moderate hypoglycemia, TRPC6

## Abstract

**Background:**

Recurrent moderate hypoglycemia (RH), a major adverse effect of hypoglycemic therapy in diabetic patients, is one of the main risk factors for cognitive impairment and dementia. Transient receptor potential canonical channel 6 (TRPC6) is a potential therapeutic target for Alzheimer's disease (AD) and its expression is highly regulated by glucose concentration.

**Objective:**

To investigate whether RH regulates the expression of TRPC6 in brain and whether TRPC6 dysfunction can drive hypoglycemia‐associated cognitive impairment in diabetes, and reveal the underlying mechanism.

**Methods:**

Histological staining, in vivo two‐photon Ca^2+^ imaging, and behavioral tests were used to measure neuronal death, brain network activity, and cognitive function in mice, respectively. High‐resolution respirometry and transmission electron microscope were used to assess mitochondrial structure and function. Intracellular calcium measurement and molecular biology techniques were conducted to uncover the underlying mechanism.

**Results:**

Here, we report that the expression of TRPC6 in hippocampus was specifically repressed by RH in streptozocin‐induced type 1 diabetic mice, but not in nondiabetic mice. TRPC6 knockout directly leads to neuron loss, neuronal activity, and cognitive function impairment under diabetic condition, the degree of which is similar to that of RH. Activation of TRPC6 with hyperforin substantially improved RH‐induced cognitive impairment. Mechanistically, TRPC6 inhibited mitochondrial fission in the hippocampus of diabetic mice undergoing RH episodes by activating adenosine 5‘‐monophosphate‐activated protein kinase, and TRPC6‐mediated cytosolic calcium influx was required for this process. Clinically, dysfunction of TRPC6 was closely associated with cognitive impairment in type 2 diabetic patients with RH.

**Conclusions:**

Our results indicate that TRPC6 is a critical sensitive cation channel to hypoglycemia and is a promising target to prevent RH‐induced cognitive impairment by properly orchestrating the mitochondrial dynamics in diabetic patients.

## Graphical abstract

Recurrent moderate hypoglycemia disturbs mitochondrial morphology and function in hippocampus by inhibiting TRPC6, leading to neuronal death and cognitive impairment in diabetic mice.

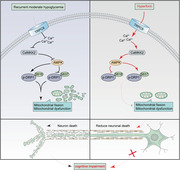

## 1 INTRODUCTION

Hypoglycemia is a common clinical event in diabetic patients, especially those with tight glycemic control using insulin or other hypoglycemic agents. As the energy source of the adult human brain almost completely depends on glucose, hypoglycemia is undoubtedly the primary risk factor for cognitive deficits in diabetic patients.^1^, ^2^, ^3^ Severe hypoglycemia (blood glucose < 2.3 mM) can lead to coma, which might cause permanent brain damage and even death. Moderate hypoglycemia, referred to blood glucose level falling to 2.3‐3.9 mM, is usually self‐remitted but not easily aware of.^4^ However, recurrent moderate hypoglycemia (RH) occurs in more than 70% patients with type 1 diabetes mellitus (T1DM) and 40% patients with type 2 diabetes mellitus (T2DM), and which is much more frequent than severe hypoglycemia.^1^, ^5^, ^6^ Unfortunately, there is no effective measure to prevent cognitive impairment induced by RH in diabetic patients at present. Hence, it is urgent to reveal the underlying mechanisms of RH‐induced cognitive deficits and identify new intervention targets.

As neuronal survival and synaptic plasticity are highly dependent on energy supply from mitochondria, neurons are extremely susceptible to mitochondrial damage compared with other cell types.^7^, ^8^ Brain mitochondrial structural and functional disorders play a critical role in cognitive impairment.^9^ The balance between mitochondrial fission and fusion is fundamental for the maintenances of morphology and function of mitochondria in response to metabolic stresses. Mitochondrial fusion, mediated by mitofusin (Mfn) 1 and 2, is thought to promote oxidative phosphorylation,^10^ whereas mitochondrial fragmentation caused by fission is associated with mitochondrial dysfunction in response to severe energy shortage.^11^, ^12^ Abnormal mitochondrial dynamics and mitochondrial dysfunction, in particular Drp1‐mediated excessive mitochondrial fission, are main steps in RH‐induced hippocampal synaptic injury and cognitive deficits.^13^ It is worth noting that mitochondrial dynamics is tightly regulated by intracellular calcium, as fusion depends on calcium oscillations^14^ and fission could be evoked by intracellular Ca^2+^ elevation accompanied with dephosphorylation of DRP1 Ser637.^15^ Therefore, RH‐induced mitochondrial dysfunction in neurons may be related to the change of intracellular calcium homeostasis.

The transient receptor potential channel 6 (TRPC6) is a Ca^2+^‐permeable nonselective cation channels,^16^, ^17^ which participate in multiple key processes in nervous system, such as neuronal survival, neural stem cell proliferation, and differentiation and synaptogenesis.^18^, ^19^, ^20^ The expression of TRPC6 is remarkably decreased in neurons of rats with cerebral ischemia^21^ and in peripheral blood leucocytes of patients with cognitive impairment.^22^ In contrast, activation of TRPC6 with hyperforin, the active constituent of St. John's Wort, significantly ameliorates the depression symptom in patients.^23^, ^24^, ^25^, ^26^, ^27^ These results support a close association between TRPC6 and cognitive function, but it is not known whether these effects are related to the regulation of cellular calcium level. In addition, compared with other TRPC members, TRPC6 displays a high sensitivity to glucose concentration as the expression and function of TRPC6 is significantly elevated by high glucose stimulation in some organs.^28^, ^29^, ^30^, ^31^ However, whether and how TRPC6 participates in RH‐induced cognitive impairment remain uninvestigated.

Here, we observed that the expression of TRPC6 in hippocampus was inhibited by RH in the case of diabetes, and TRPC6 knockout showed similar deteriorate effects on cognitive function as RH in the diabetic model. We elucidated the molecular mechanism of RH‐induced cognitive impairment and identified TRPC6 as an important target in response to hypoglycemic stimulation in the brain.

## 2 MATERIALS AND METHODS

### 2.1 Animals

TRPC6 global knockout mice (B6; 129S‐Trpc6tm1Lbi/Mmjax, stock number: 37345‐JAX, TRPC6^−/−^) and wild‐type controls were purchased from The Jackson Laboratory. Male mice (about 16‐week old) with body weight over 27 g were used and divided into the following groups: mice with STZ‐induced type 1 diabetes mellitus (DM), DM mice with recurrent moderate hypoglycemia (DM‐RH), TRPC6 deficiency mice with DM (TRPC6^−/−^‐DM), TRPC6^−/−^‐DM mice with RH (TRPC6^−/−^‐DM‐RH), nondiabetic (ND) mice, and nondiabetic mice with RH (ND‐RH). Hyperforin‐ or saline‐treated RH mice were divided into the following groups: DM‐RH, DM‐RH‐hyperforin, TRPC6^−/−^‐DM‐RH, and TRPC6^−/−^‐DM‐RH‐hyperforin.

The TRPC6^−/−^ and WT mice were received streptozotocin (STZ, 50 mg/kg/day) for 5 consecutive days to establish the type 1 diabetic model. The animals with random blood glucose > 16.7 mM defined as DM. To make sure the mice remain healthy and closely replicate the treatment in diabet, all DM mice were administrated with long‐acting insulin glargine once a day from the seventh day of STZ injection to the end of the experiment. All mice were housed according to the guidelines from Animal Ethics Committee of Daping hospital.

### 2.2 Recurrent moderate hypoglycemia episodes and hyperforin treatment

In order to induce recurrent moderate hypoglycemia attack, mice were injected with insulin (Wanbang, China, 8.0 units/kg for DM mice and 0.5‐0.8 units/kg for ND mice, i.h.) 4 hours after fasting, once every 2 days, three times a week, lasting for 8 weeks. Each injection was performed at 8 to 9 a.m. 2 hours after insulin injection, mice were feed to terminate the hypoglycemia episode. None of mice experienced with seizures or coma during RH.

Hyperforin was purchased from sigma (10 mg, PHL89225) and dissolved in DMSO (100 mg/mL), then divided and store at −80°C. The stock solution was diluted with 0.2 mL saline and injected intraperitoneally once a day (6 mg/kg/per day, PHL89225; Sigma) for 8 weeks.

### 2.3 Behavioral tests

The behavioral tests, including the Morris water maze, Y‐maze, and open field, were conducted according to previous study.^32^ To avoid the effects of acute hypoglycemia on behavioral performance, these tests were started at the fourth day after the postlast hypoglycemia exposure. All tests performed between 8:00 and 18:00. In Y‐maze test, mice first freely moved in two arms for 5 minutes, and after 2 hours, mice freely explored in three arms for 5 minutes. In open field test, mice freely moved in the apparatus for 5 minutes, and the distance traveled was recorded. The Morris water‐maze test was consisted of platform trials for 4 consecutive days and a probe trial at fifth day. Before test, one mouse in treatment group was excluded for the poor swimming ability. The escape latency and searching strategy in platform trials were measured. In probe trials, annulus crossings and the time spent in target quadrant (Q3) were analyzed.

**Highlights**

- TRPC6 expression is specifically sensitive to glucose in a concentration‐dependent manner.
- Recurrent moderate hypoglycemia impairs cognitive function by inhibiting hippocampal TRPC6.
- Repression of TRPC6 promotes hippocampal mitochondrial fission via inhibition of AMPK.
- Activation of TRPC6 by hyperforin prevents recurrent moderate hypoglycemia‐induced cognitive impairment.

### 2.4 In vivo two‐photon Ca^2+^ imaging

To directly measure cortical neuronal activity, in vivo two‐photon Ca^2+^ imaging was performed to simultaneously record spontaneous occurring somatic Ca^2+^ transients at single‐cell resolution in the prefrontal cortex (PFC). The animal surgery was performed in accordance with the standard procedure as previously described.^33^ Briefly, mice were anesthetized by inhalation of 1.8% isoflurane in gas mixture (95% O_2_ and 5% CO_2_) and maintained body temperature at 37°C. After removing the skin above the target region, a recording chamber with a hole at the front was cemented to the skull. Then, craniotomy (approximately 1.8 × 1.8 mm) was made at the skull projective point of PFC (1.7 mm lateral to middle, 2.9 mm anterior to bregma) under a dissecting microscope using a high‐speed drill. Remove the skull and keep the dura intact. Bleeding was stopped by using artificial cerebrospinal fluid (ACSF: 125 mM NaCl, 4.5 mM KCl, 26 mM NaHCO_3_, 1.25 mM NaH_2_PO_4_, 2 mM CaCl_2_,1 mM MgCl_2_ and 20 mM glucose, PH 7.4) and 1.5% agarose was added to surface of exposed cortex. The breath rate of mice maintained within 90–110 breath per minute. Neurons in layer 2/3 of the PFC (200‐250 um depth) were bulking loading with 0.5 mM highly sensitive fluorescent Ca^2+^ indicator Cal‐520^AM^ (AAT‐Bioquest, Sunnyvale, CA) under two‐photon microscope using a glass pipette.

A 4‐6 minutes consecutive recording was acquired at a 40‐Hz frame rate with a custom‐build two‐photon microscope (Suzhou Institute of Biomedical Engineering and Technology, China). The region of interest (ROI) was manually drawn, and relative fluorescence change (∆F/F) was calculated with LabVIEW 2014 (National Instruments). The morphology and time course of Ca^2+^ transients were used to excluded Glial cells signal.

### 2.5 Brain glucose and blood glucose monitoring

Mice were anesthetized by inhalation of 1.5% isoflurane. A small craniotomy (approximately 1.0 × 1.0 mm) was made. Then, the flash glucose monitoring system (FreeStyle Libre) was placed on the chamber and ensures that the probe is inserted into the brain tissue. A sensor‐matched detector used to detect brain glucose level. The blood glucose was detected by Rattail Glucose Meter.

### 2.6 Immunostaining and TUNEL staining

Animals were perfused transcardially with cold saline followed by 4% PFA. The brains were harvested and dehydrated using 45% sucrose solution. For immunostaining, 14 µm‐thick brain slices were first blocked with Immunol Staining Blocking Buffe (Beyotime, China) for 15 minutes at 37°C and then incubated with primary antibody (anti‐NeuN, anti‐MAP‐2) diluted in Primary Antibody Dilution Buffer (Beyotime) overnight at 4°C. Secondary antibody (1:300 dilution) was incubated for 2 hours at 37°C. TUNEL staining kit was used to measure neuronal apoptosis in CA1 and CA3 of hippocampus according to the manufacturer's instructions (In Situ Cell Death Detection Kit, Roche). All image was captured by using Olympus laser confocal microscope and fluorescence intensity or area fraction of positive staining was quantified by using ImageJ.

### 2.7 Oxidative stress evaluation

Mice were sacrificed by cervical dislocation. Brain was quickly extracted in ice and cut into 50‐µm‐thick sections using vibratome. Slices were incubated at 37°C in the dark with DHE (10 µM) or MitoSOX Red (5 µM) in ACSF for 45 minutes. Nuclei were visualized by incubated with DAPI solution for 30 minutes at 37°C. Images were acquired using an inverted fluorescence microscope and NIS‐Elements 3.0 software (Nikon Instruments) was used to analyze the fluorescence intensity. The malondialdehyde (MDA) level and superoxide dismutase (SOD) activity in hippocampal homogenates were measured using the MDA and SOD kits (Beyotime).

### 2.8 ELISA

Animals were sacrificed and brain was quickly harvested. Then, hippocampus was extracted by using dissecting microscope and weighted and homogenized in liquid nitrogen. The contents of serotonin (5‐HT), dopamine (DA), norepinephrine (NA), and gamma‐aminobutyric acid (GABA) in tissue homogenates were quantitatively measured by ELISA according to the manufacturer's instructions (FineTest, China).

### 2.9 Cell culture and treatment

PC12 cell lines were purchased from Procell and were cultured in 1640 medium supplemented with 10% FBS, 5% horse serum, and 100 µg/mL streptomycin/penicillin. PC12 cells were divided into NG group (5.6 mM) and HG group (25 mM) according to the d‐glucose concentration in the medium. To mimic the effect of RH in vitro, cells from NG or HG group received three times of low‐glucose (LG) treatment (LG, 3.0 mM, 12 hours per times), and defined as“NG+LG”or“HG+LG”group. During the period of LG treatment, hyperforin (5 µM, H5160; Sigma), Compound C (10 µM, MCE), AICAR (1 mM, MCE), or BAPTA‐AM (2 µM, MCE) was added separately according to the experimental target. After finishing the last LG treatment, cells were continued cultured in normal or high‐glucose medium for 4 hours and then harvested for WB test or intracellular Ca^2+^ measurement test.

Recombinant lentivirus (pLenti‐EF1a‐PuroR‐CMV‐TRPC6‐eGFP‐3xFlag), purchased from Taitool Bioscience Technology (Shanghai, China), was used for TRPC6 overexpression. si‐LKB1 or si‐CaMKK2 was used to downregulate the expression of LKB1 or CaMKK2.

### 2.10 Primary hippocampal neuron culture

Primary mouse hippocampal neurons were extracted from newborn WT or TRPC6^−/−^ mice as previously described.^34^ Briefly, hippocampus was quickly dissected and single‐cell suspension was made by using Primary Neuron Isolation Kit (88280; Invitrogen). Neurons were plated on collagen and poly‐d‐lysine‐coated dish (coring) and cultured in neurobasal media supplemented with B27 and l‐glutamine (Gibco/Thermo Fisher Scientific). Cytosine arabinoside (Sigma, 5 µM) was used to inhibit the proliferation of glial cells 3 days after plating. Thereafter, one‐third of the medium was replaced with fresh neuronal maintenance medium every 4 days. About 21 day after plating, neurons were harvested for intracellular Ca^2+^ measurement.

### 2.11 Mitochondria isolation

To assess the mitochondrial respiratory function, we extracted the mitochondria using Tissue Mitochondria Isolation Kit (Beyotime) as previously reported.^35^ Specifically, mice were sacrificed, and hippocampus was quickly dissected with a microscope. Then, the tissue was homogenized with a Dounce type glass homogenizer. The homogenates were centrifuged at 1200 *g* for 10 minutes followed by centrifuging at 3500 *g* for 10 minutes, and the remnants were collected.

### 2.12 High‐resolution respirometry and ATP determination

The mitochondrial respiratory function was determined using the Oxygraph‐2k (Oroboros Instruments, Innsbruck, Austria) with a designed substrate‐uncoupler‐inhibitor titrations (SUIT) protocols.^35^ Briefly, glutamate (10 mM) and malate (2.5 mM) were used to measure the respiratory leak state of complex I (CI Leak). The oxidative phosphorylation of complex I (CI OXPHOS) was then measured by titrating of ADP (10 mM). Succinate (1.0 mM) was subsequently titrated to confirm maximal OXPHOS capacity. FCCP was then administrated to obtained the maximal uncoupled respiratory capacity of the electron transfer system (ETS, CI+II ETS). The capacity of Complex II ETS (CII ETS) was determined by the addition of rotenone (0.5 µM). Residual oxygen consumption was last evaluated by antimycin A (Ama, 1.0 µM). ATP level in brain was determined using the Enhanced ATP Assay Kit (Beyotime).

### 2.13 Transmission electron microscope examination

The morphology of mitochondria was assessed by using a transmission electron microscope (TEM). Small fragments of the hippocampus (CA1 region, 1 mm^3^) were fixed with 3% (v/v) buffered glutaraldehyde, followed by 1% (v/v) osmium tetroxide. After being dehydrated, three to five sections per sample were examined under TEM. Images of each section were obtained, thus yielding 80‐120 mitochondria per sample for morphometry assay analysis.

### 2.14 Intracellular free Ca^2+^ measurement

Cells were loaded with Ca^2+^ indicator (Fura 2‐AM, 2 µM; Invitrogen) and 0.02% Pluronic F‐127 in the dark for 60 minutes. Then, cells were washed three times and suspended. Fluorescence intensity was calculated using a fluorescent plate reader with emission wavelength at 510 nm and excitation wavelengths of 340 and 380 nm. After the baseline measurement (25s for Ca^2+^ measurement), CaCl_2_ (1 mM) and/or hyperforin (10 µM; Sigma) and/or SAR7334 (100 nM) and/or Ruthenium red (10 µM) were added immediately and followed by 50s continuous Ca^2+^ measurement. The changes in intracellular calcium [Ca^2+^]c were calculated as the ratios of fluorescence intensity at 340 and 380 nm.^36^

### 2.15 Real‐time PCR

RNA was extracted with TRIzol (Invitrogen) and cDNA M‐MuLV Reverse Transcriptase was used to produce cDNA (New England Biolabs). QuantiTect SYBR Green RT‐PCR Kit (QIAGEN) was used for the amplification according to the three‐step protocol as described by the manufacturer.^37^ The fluorescence curves were analyzed with LightCycler 96 software.

### 2.16 Western blot

Samples were homogenized in buffer containing 0.5 mol/L Tris, 1% NP40, 1% Triton X‐100, 1 g/L sodium dodecyl sulfate, 1.5 mol/L NaCl, 0.2 mol/L EDTA, 0.01 mol/L EGTA, and protease inhibitor and/or phosphatase inhibitor (Roche). These samples were sonicated and centrifuged at 12 000 *g* for 20 minutes at 4°C. The supernatant was collected and the protein concentration was determined by the Bradford method and then 50 µg protein was loaded on 10% SDS polyacrylamide gel.^38^ Ponceau S solution was used to showed total protein. The primary antibodies were used, including anti‐TRPC1 (ACC‐010, 120 kDa), anti‐TRPC3 (ACC‐016, 100 kDa), anti‐TRPC4 (ACC‐018, 97 kDa), anti‐TRPC5 (ACC‐020, 100 kDa), anti‐TRPC6 antibody (ACC‐017, 110 kDa), anti‐TRPC7 antibody (ACC‐066, 95 kDa), anti‐TRPV1 (ACC‐030, 90 kDa), and anti‐TRPV4 (ACC‐034, 100 kDa) from Alomone; anti‐LKB1 (PA5‐96062, 48 kDa), anti‐phospho‐CaMMK2 (PA5‐105225, 65 kDa), and anti‐phospho‐DRP1 (S616, PA5‐64821, predicted to react with Ser585 in rats and Ser600 in mice, 83 kDa) antibodies from Invitrogen; anti‐CaMKK2 (ab135979, 65 kDa), anti‐Drp1 (ab184247, 83 kDa), anti‐phospho‐DRP1 (S637, ab193216, predicted to react with Ser656 in rats and Ser616 in mice, 83 kDa), anti‐PSD95 (ab238135, 80 kDa), anti‐Synapsin I (ab254349, 74 kDa), and anti‐SYP (ab32127, 34 kDa) antibodies from Abcam; anti‐AMPKα (2532S, 62 kDa) and anti‐phospho‐AMPKα (Thr172, 2535S, 62 kDa) antibodies from CST, followed by incubation with the secondary antibodies (ZSGB‐BIO). Protein expression was normalized to GAPDH or total protein.

### 2.17 Clinical study

All patients were provided with written and informed consent prior to inclusion in the study. To examine the effect of RH on cognitive function in diabetes patients, we recruited 16 type 2 diabetic (T2DM) patients with RH, 22 T2DM patients, and 18 age‐matched participants from Hospital between January 2018 and June 2019. A T2DM patient with at least three hypoglycemic events per week or total more than six hypoglycemic events within 2 weeks was considered to have RH. A hypoglycemia event was defined as blood glucose lowring than 3.9 mmol/l and sustained more than 30 min. The hypoglycemic events were detected by using the FreeStyle Libre Pro System that records up to 2 weeks of continuous glucose data. Those diabetic patients had received training for using the system from professional staff before recording. The characteristics of the participants are shown in Table S1. The cognition of subjects was assessed by using scales, including Rey Auditory Verbal Learning (RAVL), Long Delay Free Recall (LDFR), Recognitive Verbal Learning (RVL), Memory and Execution Screening‐Total (MES‐T), Memory and Execution Screening‐Memory (MES‐M), and Memory and Execution Screening‐Execution (MEX‐E).

Eight T2DM patients with RH, 18 T2DM patients without, and 14 age‐matched participants from above subjects provided their blood. The whole blood cells of each participant were collected in two EDTA tubes. One month before blood samples were collected, and the participants had not used any drugs other than insulin. White blood cells were extracted according to the following protocol. Whole blood samples were centrifuged at 500 *g* for 5 minutes and the supernatant was discarded. Then, a 10‐fold volume of ACK lysis buffer (Beyotime) was added for 5 minutes and centrifuged at 500 *g* for 5 minutes, and the supernatant was discarded. The pellet was washed two times with PBS. One of the sample was immediately stored at −80°C for TRPC mRNA quantitative analysis, and the other was used to measure TRPC6‐mediated intracellular Ca^2+^ uptake with hyperforin (Sigma) according to Xiong's method.^36^

### 2.18 Statistical analysis

In animal experiments, data are expressed as the mean ± SEM. Statistical differences between groups were assessed by Student's *t*‐test and one‐way or two‐way analysis of variance with Bonferroni's multiple comparison post hoc tests, as appropriate. In clinical study, results were presented as the mean ± SD. Baseline characteristics of the subjects were compared using the *χ* ^2^ test for categorical variables and the two‐sample *t*‐test for continuous variables. All analyses were conducted using SPSS 17.0, or GraphPad Prism software, version 6.0 (GraphPad Software). Two‐sided *P* values less than .05 were regarded to be statistically significant.

### 2.19 Study approval

All animal experiments were conducted in accordance with the Third Military Medical University Animal Welfare Committee. Clinical study was conducted in accordance with the Guide for the Clinical Investigation in patients of the Ethics Committee in Daping Hospital.

## 3 RESULTS

### 3.1 TRPC6 is an LG‐sensitive channel in brain of diabetic mice

**FIGURE 1:**
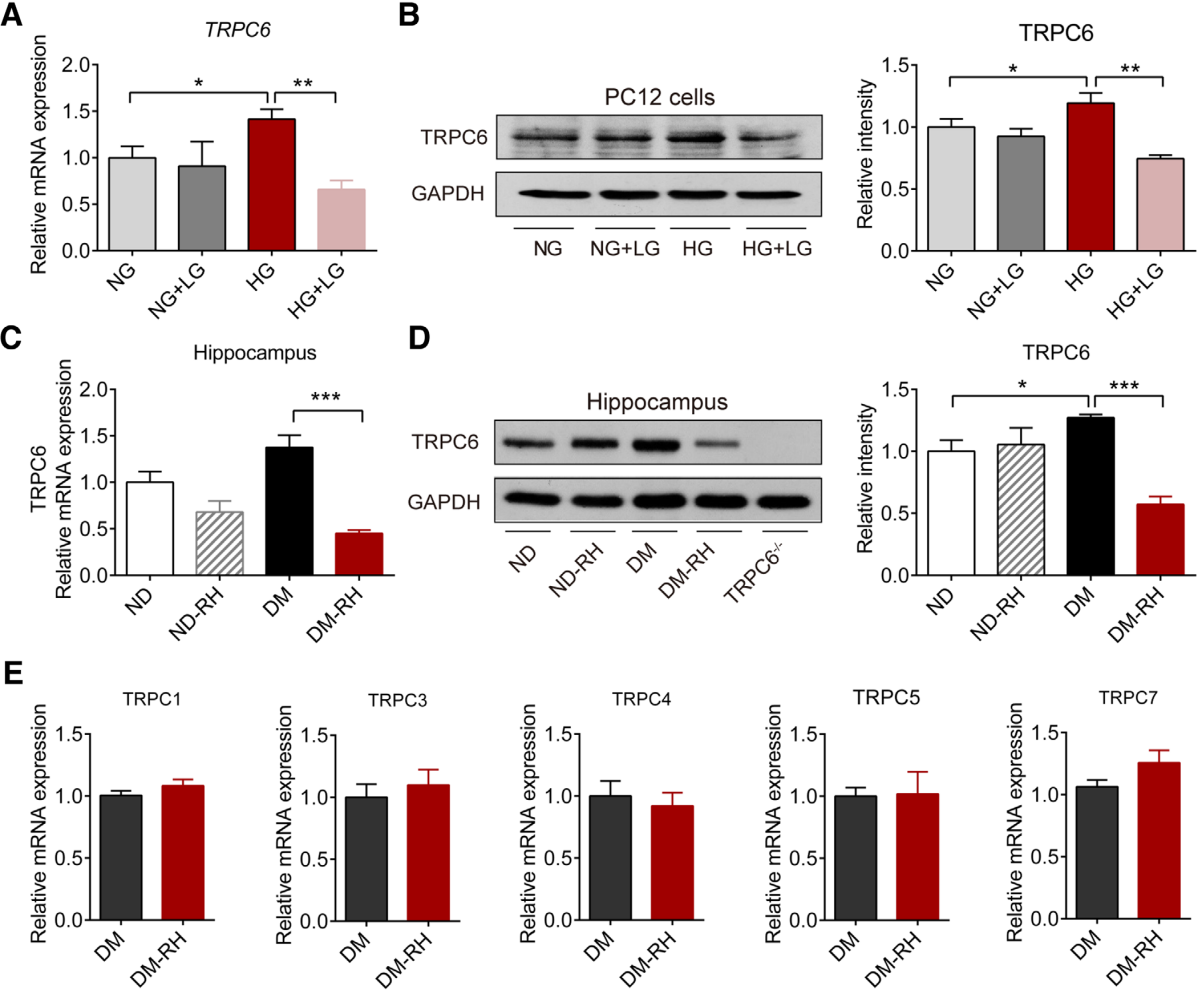
RH specifically represses hippocampal TRPC6 channel in diabetic mice. (A) Relative TRPC6 mRNA expression (n = 6) in PC12 cells cultured in medium with normal glucose concentration (NG, 5.6 mM) or medium with high glucose concentration (HG, 25 mM). NG+LG, cells cultured in NG medium and received three times of low glucose stimulation (12 hours per times, LG, 3.0 mM); HG+LG, cells cultured in HG medium and received three times of low glucose stimulation (12 hours per times, LG, 3.0 mM). (B) Representative Western blots of TRPC6 protein in PC12 cells in four groups. GAPDH served as a loading control. Quantitative data were shown on the right (n = 4). (C) Relative mRNA expression levels of TRPC6 in hippocampus from nondiabetic (ND) mice, ND mice with recurrent moderate hypoglycemia (ND‐RH), mice with STZ‐induced type 1 diabetes mellitus (DM), and DM mice with RH (DM‐RH) (n = 6 samples from six mice). (D) Representative Western blots of TRPC6 in hippocampus. GAPDH served as a loading control. Quantitative results are shown on the right (n = 3 tissues from three mice). TPRC6^−/−^, TRPC6 global knockout mice. (E) Relative mRNA expression levels of TRPC1, TRPC3, TRPC4, TRPC5, and TRPC7 in hippocampus (n = 6–8 samples from four mice). *P < .05, **P < .01, ***P < .001. Statistical significance was assessed using a one‐way ANOVA or Kruskal‐Wallis test

To investigate the direct effect of glucose on TRPC6 expression, PC12 cells were cultured in medium with normal glucose (NG, 5.6 mM) or high glucose (25 mM) and received three times of LG stimulation (3.0 mM, 12 hours per times, NG+LG, HG+LG). High glucose significantly enhanced both mRNA and protein expression levels of TRPC6; however, repeated LG stimulation lowered the expression of TRPC6 when combined with HG treatment (Figure 1A and B). In consistence, the response of TRPC6 expression in hippocampus of mice with type 1 diabetes mellitus (DM) to recurrent moderate hypoglycemic (RH) episodes was similar to that in PC12 cells. Specifically, STZ‐induced hyperglycemia significantly upregulated TRPC6 expression, whereas RH specifically inhibited TRPC6 expression in DM mice but not in nondiabetic (ND) controls (Figure S1A and Figure 1C and D). The body weight, fasting glucose level, and glucose tolerance were not significantly affected by RH attack under diabetic condition (Figure S1B‐D). However, the expression of other TRPC family members, including TRPC1, 3, 4, 5, and 7, were not significantly affected by RH in diabetic mice (Figure 1E). These results indicate that TRPC6 is an LG‐sensitive cation channel in brain under hyperglycemic status.

### 3.2 Repression of TRPC6 directly causes neuronal loss and cognitive impairment in diabetic mice

**FIGURE 2:**
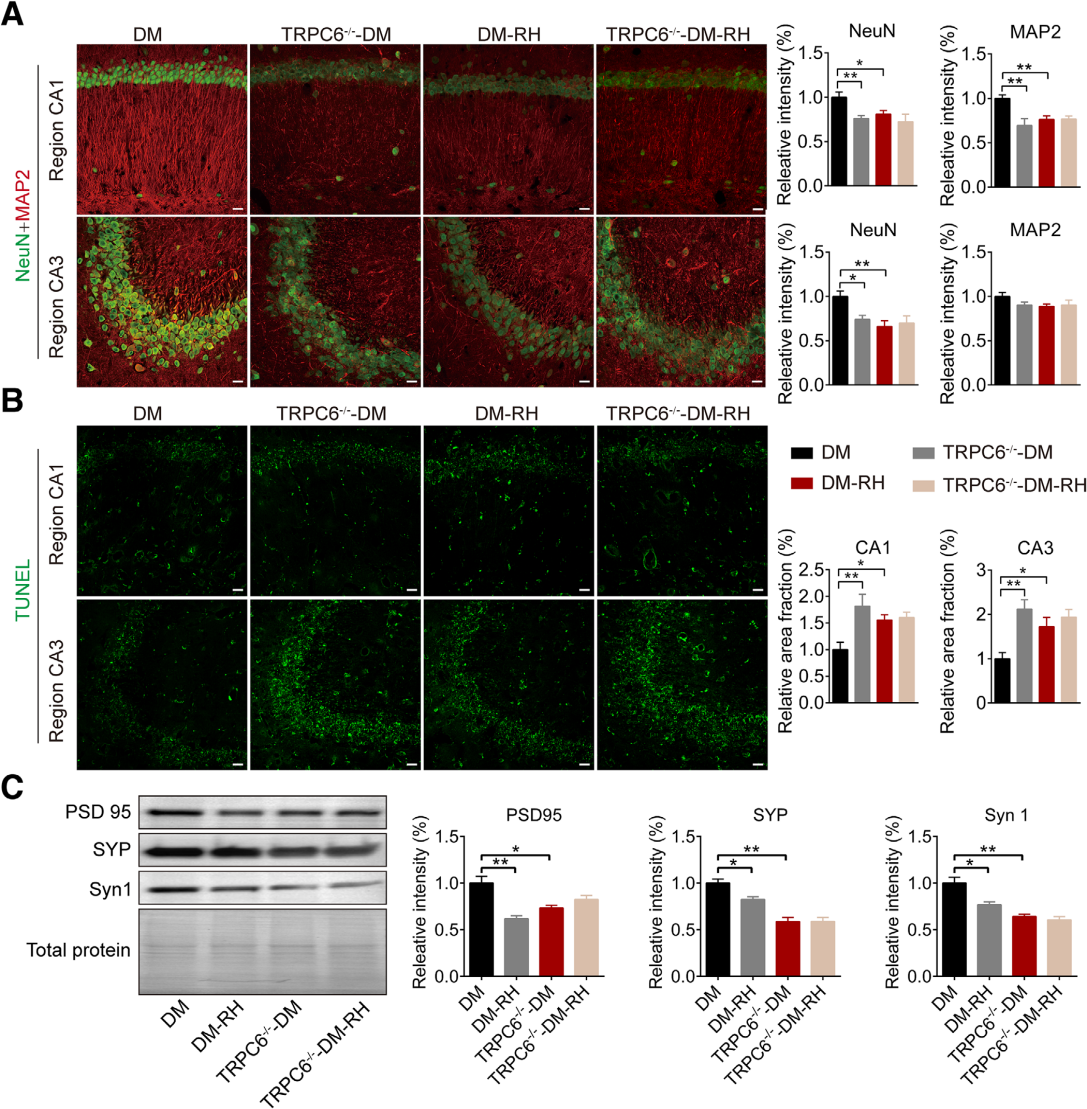
RH causes neuronal and dendritic loss in hippocampus under diabetic condition. (A) Representative image for neuronal loss detected by NeuN (neuron) and MAP2 (dendrite) immunostaining in CA1 region (*upper*) and CA3 region (*lower*) of hippocampus from indicated groups. The quantitative results showed on the right (n = 6 samples from six mice). *Scale bar*, 50 µm. DM, type 1 diabetes mellitus; DM‐RH, DM mice with recurrent moderate hypoglycemia; TRPC6^−/−^‐DM, TRPC6 global knockout mice with DM; TRPC6^−/−^‐DM‐RH, TRPC6^−/−^‐DM mice with recurrent moderate hypoglycemia episodes. (B) Representative image for neuronal apoptosis detected by TUNEL staining in CA1 and CA3 of hippocampus. Quantitative data were shown on the right (n = 6 sample from six mice). *Scale bar*, 50 µm. (C) Western blot for synapse‐associated proteins including PSD95, synaptophysin (SYP), and Synapsin I (Syn I) in hippocampal homogenates (n = 3 samples from three mice). Total protein, the amount of loaded protein detected by Ponceau S. *P < .05, **P < .01. Statistical significance was assessed using a one‐way ANOVA or Kruskal‐Wallis test

Next, to determine the role of TRPC6 repression on hippocampal morphology, neuronal network activity, and cognitive function, TRPC6 knockout mice with type 1 diabetes mellitus (TRPC6^−/−^‐DM)^39^ were challenged by RH (TRPC6^−/−^‐DM‐RH). Compared with DM mice, TRPC6^−/−^‐DM mice and DM‐RH mice showed significant decreased fluorescence intensity of NeuN (neurons) and MAP2 (dendrites) immunostainings and increased apoptosis detected by TUNEL staining in the CA1 and CA3 region of hippocampus (Figure 2A and B). Consistently, synapse‐associated protein expressions, including PSD‐95, SYP, and Syn1, were also decreased in hippocampus by TRPC6 knockout and/or RH (Figure 2C).

**FIGURE 3:**
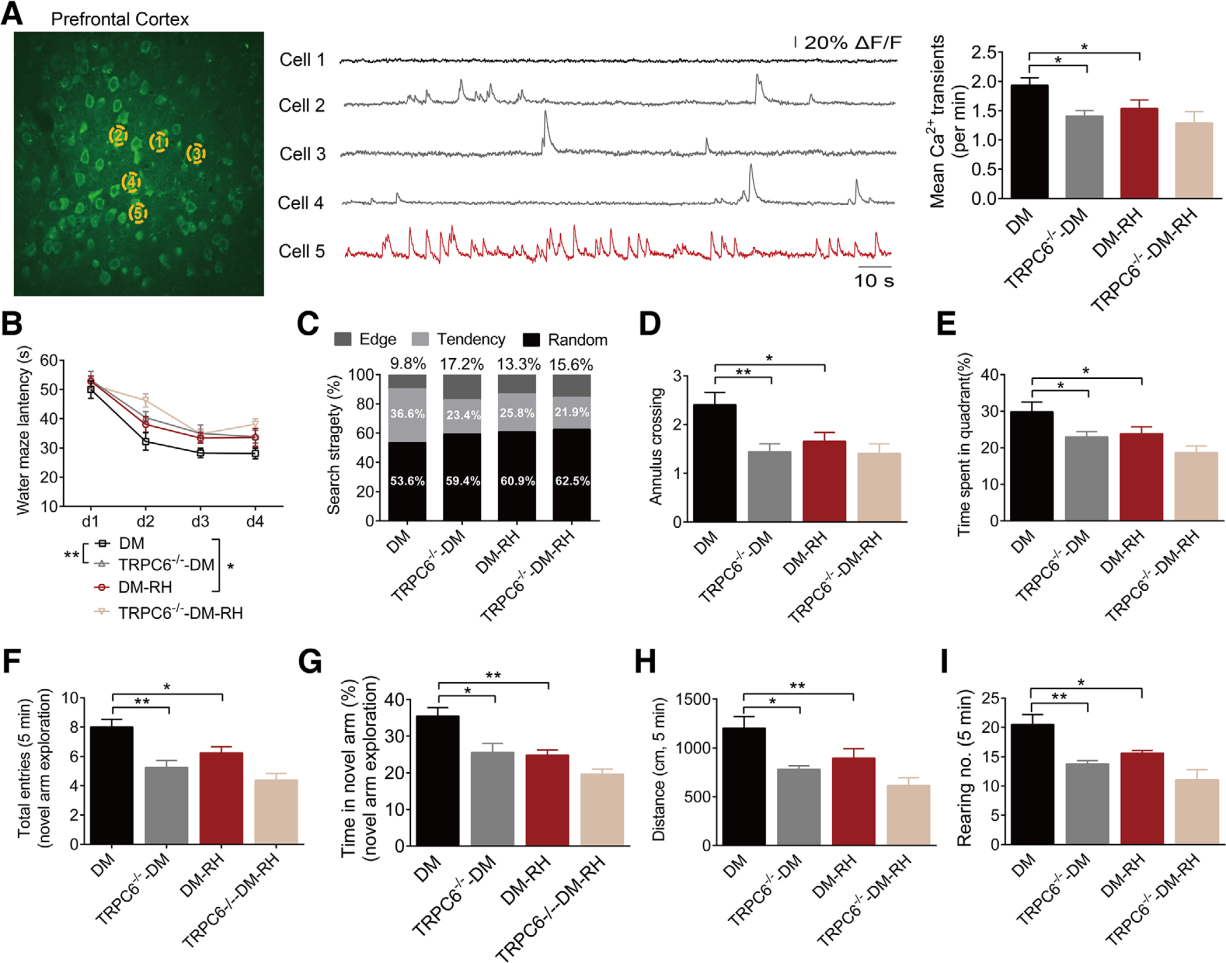
TRPC6 knockout impairs neuronal activity and behavioral performances as RH in diabetic mice. (A) Representative image showing the neuronal activity in layer 2/3 of the prefrontal cortex (PFC), assessed by in vivo two‐photon imaging to continuous recording (4‐6 minutes) spontaneous somatic Ca^2+^ transients. Traces on the right in different colors represent the three types of neurons marked in picture. Black, silent neurons (showing no transients over recording periods); gray, normal neurons (0‐4 transients per min); red, hyperactive neurons (>4 transients per min). The quantitative results showing the neuron activity recorded in four groups are on the right (n = 6–8 mice). (B‐I) The results of the behavioral tests, including the Morris water‐maze test, Y‐maze test, and open field test (n = 8–10 mice). (B, C) Escape latency and searching strategy to find the hidden platform during platform trials of the Morris‐water maze test. *Tendency*, the best strategy for mice to find the platform; *Edge* and *Random*, the poor strategy used for mice to find the platform. (D, E) The numbers of passing through the annulus and time spent in the target quadrant where the former platform located in during the probe trial of the Morris water‐maze test. (F and G) Total entries and percentage of time spent in the novel arm during the novel arm exploration trials of the Y‐maze test. (H and I) Distance traveled and rearing numbers in the open field test. The data in all the panels are expressed as the mean ± SEM. *P < .05, **P < .01. Statistical significance was assessed using a one‐way ANOVA or two‐way ANOVA by Dunnett's multiple comparisons test

Surprisingly, despite TRPC6 knockout and/or RH did not significantly changed the proportion of silent neurons, normal neurons, and hyperactive neurons (Figure S2A‐D), it significantly reduced the neuronal activity of the PFC, as evidenced by the decreased frequency of spontaneous occurring somatic Ca^2+^ transients detected by in vivo two‐photon Ca^2+^ imaging, which is similar to the effect of RH in diabetic mice (Figure 3A). Accordingly, knockout of TRPC6 led to serious decline in spatial learning, memory, and exploration ability of diabetic mice in several behavioral tests, including Morris water‐maze test (Figure 3B‐E), Y‐maze test (Figure 3F and G), and open field test (Figure 3H and I), to an almost equal extent to RH group. In addition, RH failed to the further exacerbate the above‐mentioned parameters in diabetic TRPC6^−/−^ mice (Figure 3A‐I), suggesting that the repressed TRPC6 expression was responsible for the detrimental effect of RH on cognitive deficits. Under nondiabetic condition, RH showed no significant impacts on the neuronal activity (Figure S3A‐D) and performances in behavioral tests just as it did not reduce the expression of brain TRPC6, while TRPC6 knockout caused mild impairment of performances in behavioral tests (Figure S4A‐H), further implying that repressed TRPC6 was necessary for the RH‐induced cognitive impairment. Additionally, TRPC6 gene knockout did not cause compensatory upregulation of other TRP channels in hippocampus, including TRPC1, TRPC3, TRPC4, TRPC5, TRPC7, TRPV1, and TRPV4 (Figure S5A and B).

### 3.3 Activation of TRPC6 protects against RH‐induced neuronal loss and cognitive impairment

**FIGURE 4:**
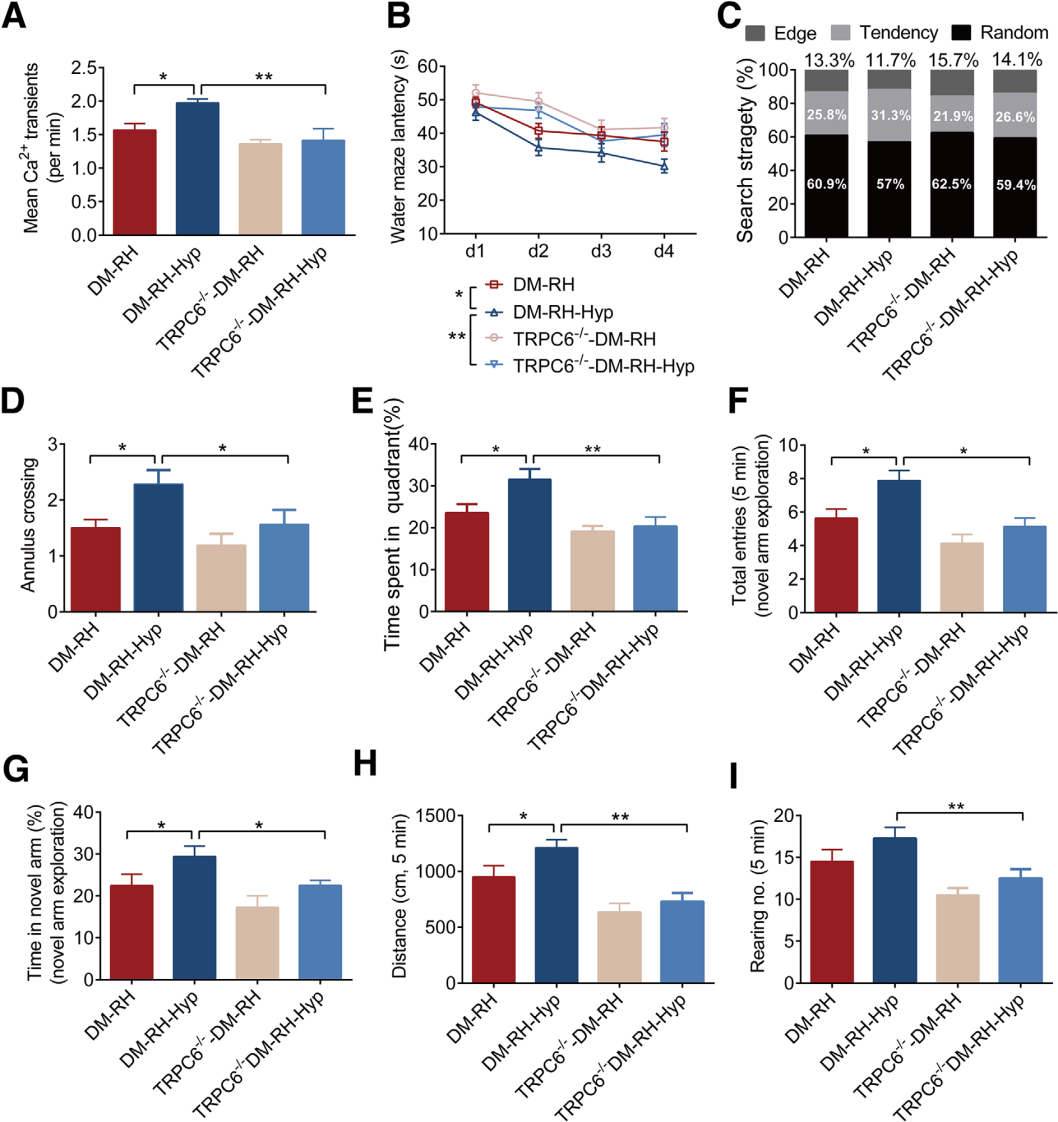
Activation of TRPC6 with hyperforin alleviates RH‐induced cognitive impairment in diabetic mice. (A) The frequency of somatic Ca^2+^ transients in PFC of mice from the four indicated groups (n = 6–8 mice). Hyp, hyperforin; (B‐E) The escape latency, searching strategy, number of passing through the annulus, and percentage of time spent in the target quadrant (n = 8–10 mice). (F and G) Total entries and percentage of time spent in the novel arm during the novel arm exploration trials of the Y‐maze test (n = 10 mice). (H and I) Distance traveled and rearing numbers in the open field test. The data in all the panels are expressed as the mean ± SEM. *P < .05, **P < .01. Statistical significance was assessed using a one‐way ANOVA or two‐way ANOVA by Dunnett's multiple comparisons test

To further determine whether the reduction of brain TRPC6 is causally related to RH‐caused cognitive impairment, diabetic mice were treated with hyperforin, the agonist of TRPC6. Activation of TRPC6 by hyperforin not only alleviated the loss of hippocampal neurons (Figure S6A‐C) and the damage of neuronal activity in DM‐RH mice (Figure 4A) but also significantly improved the behavioral performances in behavioral tests without affecting body weight, blood glucose, and glucose tolerance (Figure 4B‐I and Figure SE‐G). Specifically, hyperforin decreased the latency to find the hidden platform and improved searching strategy used to find the platform (tendency, 31.3% in DM‐RH‐Hyp vs 25.8% in DM‐RH), increased number of annulus crossings, and time spent in correct quadrant in the water‐maze test (Figure 4B‐E). In Y‐maze test, hyperforin‐treated group showed a higher tendency to explore a novel environment as determined by more total entries and time spent to the novel arm than the control group (Figure 4F and G). The improvement of exploratory behavior by hyperforin was further proved by the open‐field test as evidenced by more distance traveled and rearing times (Figure 4H‐I). However, hyperforin was invalid to improve neuronal loss, neuronal activity, and behavioral performances in TRPC6^−/−^ diabetic mice (Figure 4A‐I), indicating that the protective effect of hyperforin against RH‐induced cognitive deficits was dependent on TRPC6. In addition, hyperforin treatment did not significantly alter the proportion of three types of neurons in PFC in DM mice (Figure S7A‐D) and had no impact on the cognition in ND mice ( Figure S4A‐H).

As hyperforin has additional effects except TRPC6 activation, such as inhibiting synaptosomal reuptake of neurotransmitter, we measured the amount of 5‐HT, DA, NA, and GABA in hippocampal homogenates. The results showed that the levels of 5‐HT, DA, NA, and GABA were not significant changed in DM‐RH and TRPC6^−/−^‐mice, suggesting that the cognition impairment caused by RH may not likely related to these neurotransmitters (Figure S8A). However, hyperforin treatment significantly increased the levels of DA and 5‐HT, and these effects also presented in TRPC6^−/−^ mice (Figure S8B), indicating that hyperforin exerts additional effects in a TRPC6‐independent manner.

### 3.4 RH causes excessive mitochondrial fission in brain of diabetic mice via repressing TRPC6

**FIGURE 5:**
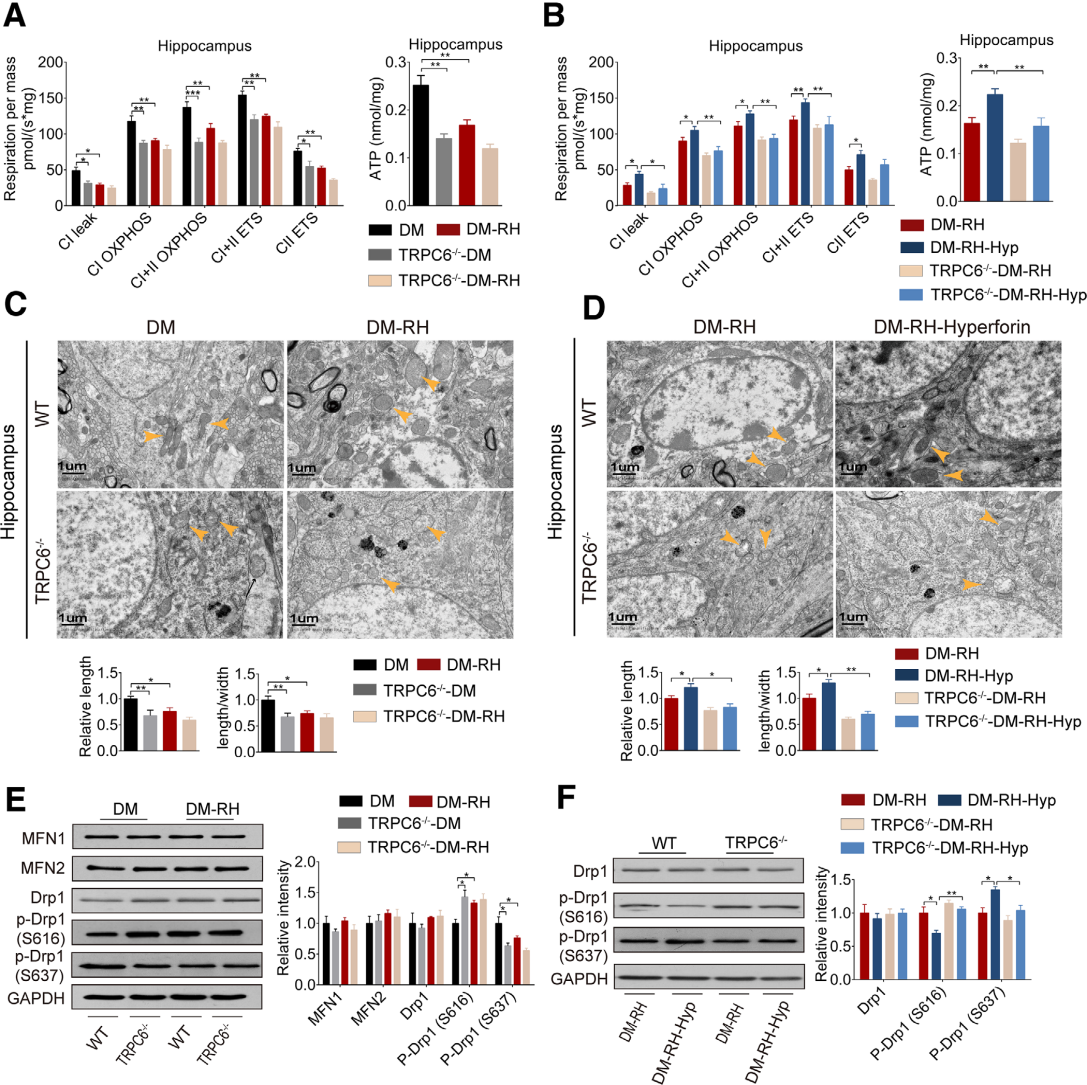
RH impairs mitochondrial function and promotes excessive Drp1‐mediated fission via repressing TRPC6 in diabetic mice. (A) Summarized data for the oxygen consumption capacity of hippocampal mitochondria measured by high‐resolution respirometry (left) and ATP content (right) of hippocampus from diabetic WT or TRPC6^−/−^ mice with or without RH (n = 6 tissues from mice). CI Leak, leak respiration of CI; CI OXPHOS, CI + CII OXPHOS, CI, and CI plus CII oxidative phosphorylation capacity; CI ETS, CI + CII ETS, CI, and CI plus CII electron transfer system capacity; CI ETS, CI + CII ETS, CI, and CI plus CII electron transfer system capacity. (B) Summarized data for the oxygen consumption capacity of mitochondria (left) and ATP content (right) of hippocampal from hyperforin or vehicle‐treated diabetic WT or TRPC6^−/−^ mice with RH (n = 6 tissues from 6 mice). (C and D) TEM images of mitochondria (indicated by yellow arrows) in the CA1 region of hippocampus. Quantitative data of mitochondrial length and ratio of length to width are shown below (n = 80–120 mitochondria from three mice). Scale bar, 1 µm. (E, F) Western blots of MFN1, MFN2, Drp1, p‐Drp1 (S616), and p‐Drp1 (S637) in hippocampus homogenates of mice. Quantitative data are shown on the right (n = 4 tissues from four mice). *P < .05, **P < .01, ***P < .01 (two‐way or one‐way ANOVA or Kruskal‐Wallis test)

As mitochondrial function plays a critical role in the maintenance of normal neuronal activity, we detected the activity of oxidative phosphorylation chain in isolated mitochondrion from the hippocampus of mice using high‐resolution respirometry. As expected, similar to RH attack, TRPC6 knockout resulted in significant hippocampal mitochondrial dysfunction in diabetic mice, as reflected by the impaired mitochondrial function, including leak respiration of CI, CI, and CII oxidative phosphorylation capacity, and electron transfer capacity, accompanied by decreased mitochondrial ATP synthesis (Figure 5A). Also, RH failed to further dampen the impaired mitochondrial function in TRPC6^−/−^ diabetic mice (Figure 5A). In contrast, hyperforin or overexpression of TRPC6 significantly alleviated RH or repeated LG‐induced mitochondrial dysfunction and ATP synthesis in a TRPC6‐dependent manner, respectively (Figure 5B and Figure S9A and B). Consistently, results from TEM revealed that TRPC6 knockout and or RH caused marked mitochondrial swelling and fragmentation as reflected by lowering of the mitochondrial length (major axes) and ratio of length to width in the CA1 region of hippocampus, and which were significantly improved by hyperforin (Figure 5C and D). The hippocampal mitochondrial morphology and function was not impaired by RH in nondiabetic mice (Figure S9C‐E). Mitochondrial fragmentation and swelling are closely associated with excessive oxidative stress.^40^ We found that DHE and MitoSox Red fluorescence intensity in hippocampus was significantly increased in TRPC6^−/−^‐DM mice and DM‐RH mice (Figure S10A and B), accompanied with increased MDA level and reduced SOD activity (Figure S9E and F). Hyperforin treatment also decreased oxidative stress level in hippocampus caused by RH in a TRPC6‐dependent manner (Figure S10C, D, G, and H).

Analyzing the expression of some molecules related to mitochondrial dynamics showed that TRPC6 knockout or RH attack significantly promoted phosphorylation of Drp1 on S616 site but reduced phosphorylation on S637 site, implying an elevated mitochondrial fission (Figure 5E and F). And these changes were almost erased by hyperforin. Other molecules, including total‐Drp1 (t‐Drp1), MFN1, and MFN2, were not significantly altered (Figure 5E). These results indicate that RH increased mitochondrial fission in hippocampus of diabetic mice by repressing TRPC6.

### 3.5 Inhibition of AMPK mediates RH‐induced excessive mitochondrial fission by TRPC6 repression

**FIGURE 6:**
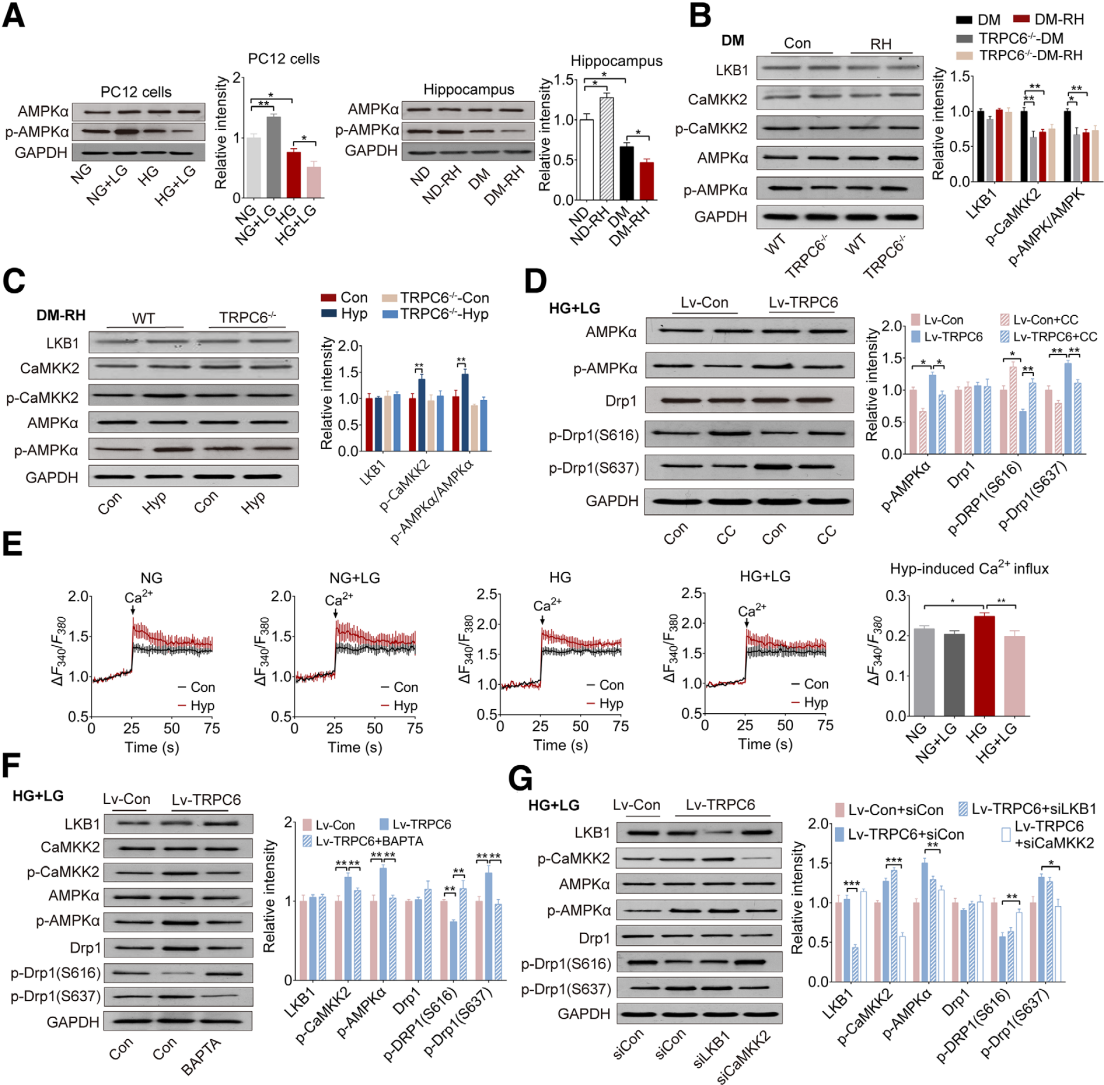
Inhibition of AMPK by TRPC6/ CaMKK2 pathway disturbs phosphorylation of Drp1. (A) Representative Western blots of AMPKα and p‐AMPKα in PC12 cells or mouse hippocampus. The quantitative data are showed on the right (n = 3 for PC12 cells, n = 4 tissues from four mice). (B) Representative Western blots of LKB1, CaMKK2, p‐CaMKK2, AMPKα, and p‐AMPKα in the hippocampus of DM mice with or without RH. The quantitative data are shown on the right (n = 3 tissues from three mice). TRPC6^−/−^, TRPC6 global knockout mice. (C) Representative Western blots of LKB1, CaMKK2, p‐CaMKK2, AMPKα, and p‐AMPKα in the hippocampus of hyperforin or vehicle‐treated diabetic WT or TRPC6^−/−^‐mice with RH. The quantitative data are showed on the right (n = 3). Hyp, hyperforin. (D) The protein expression level of AMPKα, p‐AMPKα, Drp1, p‐Drp1, (S616), and p‐Drp1 (S637) in PC12 cells treated with CC. The statistical results are showed on the right (n = 3). CC, AMPK inhibitor compound C (10 µM); Lv‐TRPC6, TRPC6 overexpression with lentivirus; Lv‐Con, the vehicle lentivirus. (E) The hyperforin (10 µM) evoked intracellular Ca^2+^ influx in PC12 cells cultured. The quantitative data are showed on the right (n = 3). TRPC6‐mediated Ca^2+^ influx was determined by the subtraction of hyperforin‐ and Ca^2+^‐induced Ca^2+^ influx to Ca^2+^‐induced influx. Ca^2+^ (1 mM) and/or hypforin (10 µM) was added at 25S. Ca^2+^, Cacl_2_ (1 mM); Hyp, hyperforim (10 µM). (F) The protein levels of LKB1, CaMKK2, p‐CaMKK2, AMPKα, p‐AMPKα, Drp1, p‐Drp1 (S616), and p‐Drp1 (S637) in PC12 cells treated with BAPTA. The quantitative data are showed on the right (n = 3). BAPTA‐AM (2 µM), intracellular calcium chelating agent. (G) The protein levels of LKB1, CaMKK2, p‐CaMKK2, AMPKα, p‐AMPKα, Drp1, p‐Drp1 (S616) and p‐Drp1 (S637) in PC12 cells with LKB1 or CaMKK2 silence by siRNA (n = 3). siCon, control siRNA. GAPDH served as a loading control. *P < 0.05, **P < 0.01 and ***P < 0.01. Statistical significance was assessed using a onexs‐way ANOVA followed by Dunnett's multiple comparisons test or Kruskal‐Wallis test followed by Dunn's multiple comparisons test

As Drp1 phosphorylation is regulated by AMP‐activated protein kinase (AMPK), an energy sensor regulating mitochondrial fission,^12^, ^41^, ^42^ we detected the phosphorylation level, the active form of the main catalytic α subunit of AMPK in PC12 cells or mouse brain (cortex and hippocampus). It showed that the phosphorylation of AMPKα (Thr172) was remarkably reduced by repeated LG stimulation or RH attack under HG or diabetic status, respectively, but not under normal condition (Figure 6A). Correspondingly, the phosphorylation of an upstream regulator of AMPK, Ca^2+^/calmodulin‐dependent protein kinase kinase (CaMKK) 2, but not LKB1, was reduced by TRPC6 knockout or RH attack in hippocampus of diabetic mice, accompanied by reduced phosphorylation of AMPKα (Figure 6B). Intervention by hyperforin also increased the phosphorylation of AMPKα and CaMKK2 in wild‐type (WT) mice receiving RH challenge but not in TRPC6^−/−^ mice (Figure 6C). Moreover, lentivirus‐mediated TRPC6 overexpression remarkably increased the phosphorylation of AMPK in PC12 cells challenged by LG stimulation, accompanied by reduced mitochondrial fission, as indicated by lower phosphorylation level on S616 but higher on S637 site of Drp1 (Figure 6D). These beneficial effects of TRPC6 overexpression were almost totally erased by treatment with compound C (CC) (Figure 6D), a potent inhibitor of AMPK. Additionally, activating AMPK with AICAR partially blocked the effects of repeated LG stimulation or TRPC6 downregulation with siRNA on the phosphorylation of Drp1 at serine 616 and serine 637 (Figure S11A and B), indicating the necessity of AMPK in the protective effect of TRPC6 against excessive mitochondrial fission evoked by RH.

### 3.6 Repression of TRPC6‐mediated Ca^2+^ influx is responsible for AMPK inactivation

Further, to determine whether TRPC6‐mediated Ca^2+^ influx was critical to activation of AMPK, we measured intracellular Ca^2+^ level in PC12 cells by monitoring the Fura‐2 immunofluorescent signal. PC12 cells cultured in HG medium displayed a remarkable higher and persistent Ca^2+^ influx as reflected by increase in Ca^2+^‐stimulated cytosolic Ca^2+^ elevation and store‐operated Ca^2+^ entry, which were not affected by LG stimulation (Figure S12A and B). We then demonstrated that hyperforin‐induced cytosolic Ca^2+^ transients were TRPC6‐mediated extracellular Ca^2+^ entry. Specifically, hyperforin‐induced Ca^2+^ influx in PC12 cells were significantly blocked by SAR7334 (an inhibitor of TRPC6) or siRNA‐mediated TRPC6 knockdown but not significantly altered by ruthenium red (Figure S12C‐E). Moreover, hyperforin‐induced cytosolic Ca^2+^ transients were completely abolished in the primary hippocampal neurons from TRPC6^−/−^ mice but not in WT mice (Figure S12F). Repeated LG challenge induced a significant decrease of TRPC6‐mediated Ca^2+^ influx in PC12 cells cultured in HG condition, but was not in NG condition (Figure 6E). Accordingly, TRPC6 overexpression increased the phosphorylation of CaMKK2 and AMPK without affecting LKB1 expression, accompanied with decreased phosphorylation on S616 (Figure 6F). However, blocking intracellular Ca^2+^ by BAPTA remarkably lowered the phosphorylation of CaMKK2 and AMPK, resulting in increased phosphorylation on S616 and decreased phosphorylation on S637 of Drp1, indicating that TRPC6‐mediated transient Ca^2+^ influx was critical for the recovery of AMPK activity in cells receiving repeated LG stimulation (Figure 6F). In addition, siRNA‐mediated knockdown of CaMKK2, but not LKB1, significantly blocked the promotional effect of TRPC6 overexpression on activation of AMPK, leading to increased mitochondrial fission compared with si‐con group (Figure 6G). These results confirmed that repeated LG stimulation reduced AMPK activity and enhanced mitochondrial fission by inhibiting TRPC6‐mediated Ca^2+^ influx. Taken together, RH induces excessive mitochondrial fission and mitochondrial dysfunction in hippocampus through inhibiting TRPC6/Ca^2+^/AMPK pathway, which inceases Drp1 phosphorylation on Ser616 and decreases it on Ser637, thus leading to neuronal death and cognitve impairment in diabetic mice (Figure 8).

### 3.7 Cellular TRPC6 dysfunction is associated with RH‐caused cognitive impairment in type 2 diabetic patients

As TRPC6 may be a probe for the early impairment of cognition,^22^, ^43^ we collected white blood cells from some of the type 2 diabetic patients with or without RH. The basic demographic information of these patients is shown in Table S1. According to the statistical results of cognitive scale (Table S2), RH is mainly related to the memory loss of diabetic patients, but does not affect their executive ability (Table S2), suggesting that hippocampus is the most vulnerable area response to RH episodes.

**FIGURE 7:**
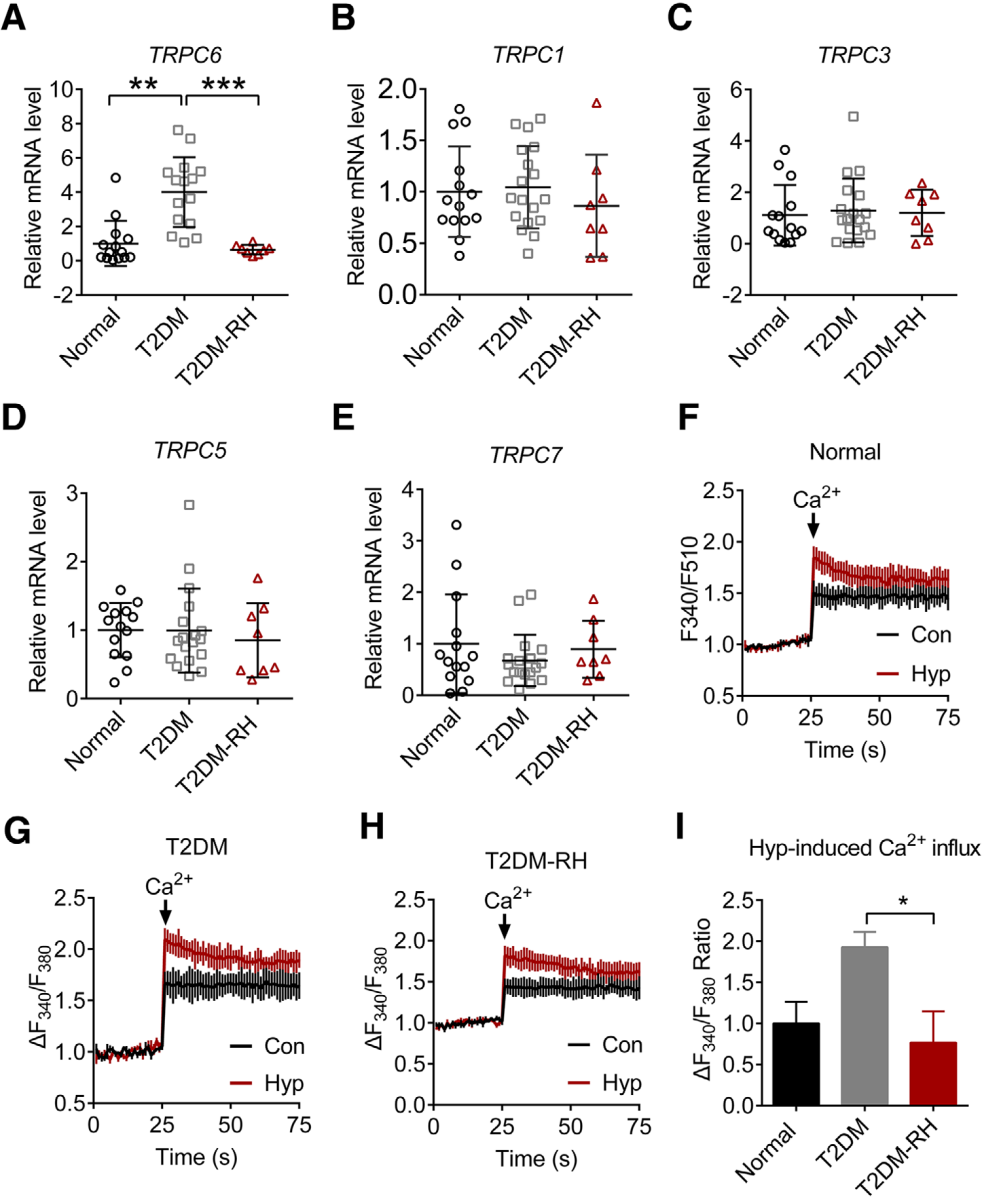
RH specifically reduces TRPC6 expression in the white blood cells from diabetic patients. (A) Relative mRNA expression of TRPC6 in human blood white blood cells from normal subject (n = 14 samples from 14 patients), type 2 diabetic patients without hypoglycemia episodes (T2DM, n = 18 samples from 18 patients) or diabetic patients with RH (T2DM‐RH, n = 8 samples from eight patients). (B‐E) Relative mRNA expression of TRPC1, TRPC3, TRPC5, and TRPC7 in human white blood cells. (F‐I) Representative image showed Ca^2+^ influx in white blood cells from a normal subject (F), a T2DM patient without RH (G), and a T2DM patient with RH and quantitative data are shown on the right (I). Ca^2+^ (1 mM, Cacl_2_) and/or hyperforin (Hyp, 10 µM) was added at the point 25S; *P < 0.01, **P < 0.01, and ***P < 0.001. Statistical significance was assessed using Kruskal‐Wallis test followed by Dunn's multiple comparisons test

The mRNA expression level of TRPC channels in white blood cells of fasting venous blood samples were collected from eight type 2 diabetic patients with RH, 18 type 2 diabetes patients without RH, and 14 age‐matched normal controls. We found that the mRNA expression of TRPC6 was significantly reduced in white blood cells of diabetic patients with RH compared with those without RH (Figure 7A). However, other TRPC members, including TRPC1, TRPC3, TRPC5, and TRPC7, were not affected (Figure 7B‐E). Accordingly, the white blood cells of diabetic patients with RH displayed a significant decrease of TRPC6‐mediated extracellular Ca^2+^ entry (Figure 7F). These results suggest a close association between TRPC6 expression reduction and cognitive dysfunction in diabetic patients with RH.

**FIGURE 8:**
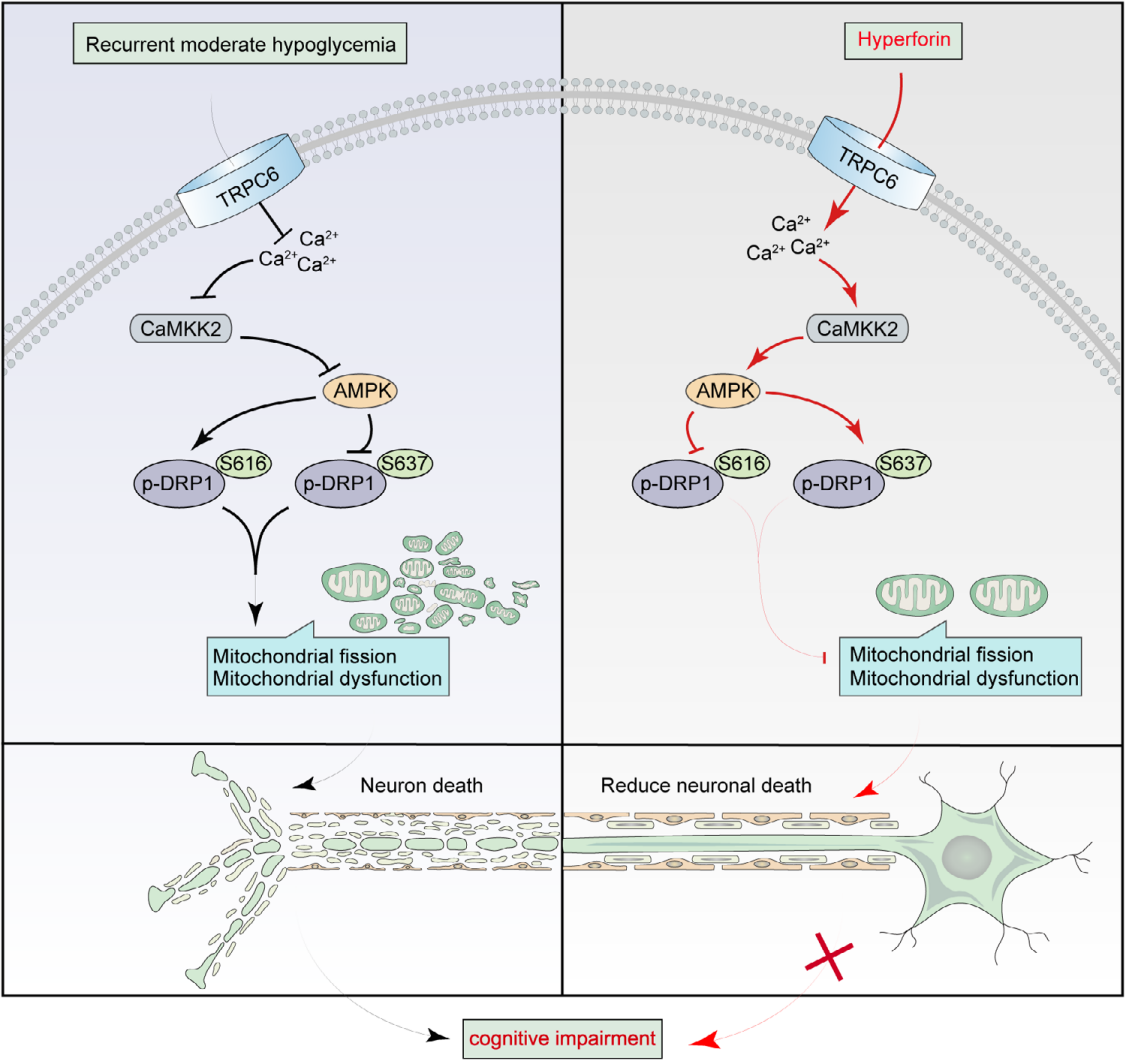
The mechanism diagram for the cognitive impairment by RH. Recurrent hypoglycemia reduces TRPC6‐mediated transient Ca^2+^ intake, which induces excessive mitochondrial fission and mitochondrial dysfunction through increasing AMPK‐dependent DRP1 phosphorylation on Ser616 and reducing it on Ser637, leading to neuronal death and cognitive impairment in diabetic mice. Treatment with hyperforin represses cognitive impairment in diabetic mice undergoing recurrent hypoglycemia by activating TRPC6

## 4 DISCUSSION

Diabetes is one of the major risk factors for dementia. Delaying the appearance of dementia phenotype can significantly reduce the burden of family and society. The determinants of the accelerated cognitive decline in diabetes, however, are less clearly recognized because diabetes is also a complex syndrome including multiple pathophysiological changes, such as chronic hyperglycemia, treatment‐induced recurrent hypoglycemia, insulin resistance, oxidative, mitochondrial dysfunction, and so on. As a major detrimental factor in hypoglycemic therapy for both T1DM and T2DM, recurrent moderate hypoglycemia (RH) is difficult to avoid in clinical practice. Despite RH has been reported to prevent age‐related decline in hippocampally cognitive function in nondiabetic rats,^44^ it exerts significant detrimental effects on cognition under diabetic condition and the mechanism remains unclear.^13^, ^45^, ^46^, ^47^, ^48^ Here, we provide experimental and clinical evidence to identify TRPC6 as a potential intervention target for RH‐induced cognitive impairment in diabetic patients. We found that the repressed TRPC6 is related to the cognitive impairment caused by RH, and the absence of TRPC6 directly led to cognitive dysfunction in STZ‐induced type 1 diabetic mice. The activation of TRPC6 by hyperforin significantly improved RH‐induced cognitive impairment and mitochondrial dysfunction and these favorable impacts were absent in TRPC6 knockout mice. The study also revealed that TRPC6‐mediated transient Ca^2+^ intake blocked RH‐induced excessive mitochondrial fission by activating AMPK. Finally, clinical data prove that TRPC6 dysfunction is associated with cognitive impairment in type 2 diabetic patients.

Transient receptor potential canonical (TRPC) channels are highly distributed in the cerebral cortex, hippocampus, cerebellum, and amygdala, where they regulate diverse neuronal and glial cell functions.^49^, ^50^ Previous studies confirmed that TRPC6 plays critical role in maintenance of neuronal activity and synaptic plasticity.^51^, ^52^ In addition, TRPC6 exerts potent neuronal protective effects against Aβ production and alleviates structural and behavioral impairment in APP/PS1 mice.^23^ However, the exact role of TRPC6 in diabetic neurological damage has not been elucidated. Previous study reported that high glucose specifically increases TRPC6 expression in podocytes, while TRPC1 and TRPC5 were unaltered.^28^ In particular, glucose enhances TRPC6 promoter activity in a concentration‐dependent manner.^28^ In endothelial cells, however, high glucose significantly increased TRPC1 expression without affecting other TRPC channels.^53^ In brain, TRPC6 expression was reduced in a rat model of stroke. Our and other's results indicate that expression of TRPC6 showed high sensitivity to glucose concentration in kidney and brain but not in endothelial cells, suggesting that there are differences in TRPC6 regulation by glucose. Unfortunately, we currently lack a clear understanding of TRPC6 regulation by glucose despite MAPK, ERK, JNK, NF‐kB, and calcineurin/NFAT pathways have been reported to participate in this process.^54^, ^55^ TRPC6 knockout directly led to cognitive impairment in diabetic mice to an extent similar to RH and RH failed to further impair cognitive function in TRPC6^−/−^ diabetic mice. Additionally, we confirmed that the expression of TRPC6 in white blood cells of diabetic patients with RH is lower than those without it and the expression of other TRPC channel were not significantly changed, but whether a consistent phenomenon could appear in neurons of diabetic patients remains uncertainly. Consistently, Lu et al also reported that TRPC6 mRNA levels in white blood cells are reduced in patients with AD and mild cognitive impairment.^22^ Thus, TRPC6 dysfunction might be a major step for cognitive impairment in diabetic patients with RH.

Moreover, activation of TRPC6 by hyperforin remarkably recovered cognitive function in diabetic mice experienced RH attack, implying that TRPC6 acts as a guardian protecting against RH‐induced cognitive impairment. It is known that hyperforin has other actions besides activating TRPC6, such as modulating of neurotransmitter, intracellular PH, upregulation of metabolizing enzymes of the cytochrome P450 family and BDNF expression, and so on.^56^, ^57^ Inhibition of synaptosomal re‐uptake of numerous neurotransmitters, such as 5‐HT, DA, NA, and GABA, is proved to be the potential mechanism for the antidepressant effects of hyperforin, which seems to be related to an elevation in intracellular Na^+^ concentration.^56^, ^58^ Thus, we have measured the levels of several neurotransmitters in the hippocampus by ELISA. As shown in Figure S8, levels of 5‐HT, DA, NA, and GABA were not significant changed in DM‐RH mice and TRPC6^−/−^‐mice, suggesting that the cognition impairment caused by RH may not likely related to these neurotransmitters. However, hyperforin treatment significantly increased the level of DA and 5‐HT, and these improvement effects also presented in TRPC6^−/−^ mice. These results suggest that hyperforin exerts additional neuronal effects in a TRPC6‐independent manner. Additionally, in this study, we found that RH and/or TRPC6 knockout caused cognitive impairments under diabetic condition, and the cognitive improvement effect of hyperforin was absent in TRPC6^−/−^ mice, indicating that TRPC6 repression by RH is critical for the cognitive impairment in diabetes. Therefore, TRPC6 is essential for the beneficial effect of hyperforin on RH‐caused cognitive impairment despite hyperforin also has other protective effects unrelated to TRPC6. It is also worth noting that TPRC6 has been reported to promote cardiac hypertrophy and progression of diabetic kidney disease through increasing Ca^2+^ entry as well as membrane depolarization.^59^, ^60^, ^61^ Thus, the heart and kidney function in diabetic patients should be carefully monitored when using hyperforin to protect their cognitive function clinically.

Mitochondria are essential for survival and excitability maintenance of neurons due to their limited glycolytic capacity.^7^, ^62^ Mitochondria in brain undergo fission or fusion process to maintain a normal morphology in response to metabolic stress such as ageing and diabetes. A recent work determined the critical role of hippocampus mitochondrial fission in the detrimental effect of RH on cognitive function in diabetic mice.^13^ Here, we demonstrated that the beneficial effect of hyperforin on RH‐induced mitochondrial dysfunction and structural damage was also relied on TRPC6 activation, further demonstrating that RH promoted hippocampus mitochondrial fission and cognitive impairment in diabetic mice by repressing TRPC6.

AMPK served as a regulator of mitochondrial fission through directly regulating Drp1 phosphorylation to maintain overall energetic homeostasis in response to energy stress.^41^, ^42^, ^63^ Although activation of AMPK is reported to promote fission by regulating phosphorylation of mitochondrial fission factor (MFF) under normal condition,^63^ it also inhibits mitochondrial fission via regulation of Drp1 expression or phosphorylation under high glucose stimulation or diabetic condition.^42^, ^64^ Thus, different disease models or cell type seems to have different impacts on AMPK‐Drp1 cascade. In this study, we confirmed that the reduced AMPK activity was critical to RH‐induced mitochondrial fission in the hippocampus of diabetic mice through impacting Drp1 phosphorylation. The AMPK activation by Ca^2+^ showed a biphasic feature, as the intermittent transient Ca^2+^ oscillation boosts AMPK activation by stimulating CaMKK2 phosphorylation, whereas sustained calcium overload decreases AMPK activity.^65^ In this study, high glucose induced permanent cytosolic Ca^2+^ uptake in PC12 cells, accompanied with decreased AMPK activity. A recent study supports that activation of AMPK not only requires a relatively lower energy status, but also relies on TRP channel‐mediated cytosolic Ca^2+^ elevation,^66^ indicating that TRP channels plays important role in regulation of AMPK activation. Here, we found that RH or repeated LG stimulation significantly reduced TRPC6‐mediated cytosolic Ca^2+^ influx, and thus further reduced AMPK activity. On the other hand, the improvement effect of TRPC6 overexpression on mitochondrial function was also relied on AMPK activation.

In summary, this study identifies TRPC6 as a previously unrecognized sensor in response to hypoglycemia in brain under diabetic condition. TRPC6 knockout showed similar detrimental effects on cortical neuron activity and cognitive function as RH, which was due to the disturbance of hippocampal mitochondrial morphology and function through inhibition of TRPC6/AMPK/Drp1 pathway. TRPC6 might be a promising target for the prevention and treatment of hypoglycemia‐related cognitive impairment in diabetic patients.

## CONFLICT OF INTEREST

The authors have declared no conflict of interest.

## AUTHOR CONTRIBUTIONS

Zhiming Zhu initiated the project. Zhiming Zhu and Chengkang He designed the experiments. Chengkang He and Peng Gao wrote the paper. Yuanting Cui, Zongshi Lu, Huan Ma, and Yu Zhao contributed to experiments. Chengkang He, Yingsha Li, Qiang Li, and Fang Sun collected and provided clinical data. Chengkang He and Li Li analyzed data. Gangyi Yang, Hongting Zheng, Xiaowei Chen, Hongbo Jia, and Daoyan Liu critically read and revised the paper. All authors read and approved the manuscript.

## Supporting information

Supplementary Figures

## Data Availability

The data that support the findings of this study are available from the corresponding author upon reasonable request.
