# Supplementary material for: Low‐glucose‐sensitive TRPC6 dysfunction drives hypoglycemia‐induced cognitive impairment in diabetes
Source: Clin Transl Med. 2020 Oct 18;10(6):e205. doi: 10.1002/ctm2.205 (PMC7568851; doi:10.1002/ctm2.205)
Supplement: Supplementary file 1 — Supplementary Figures [file CTM2-10-e205-s001.docx]

**SUPPLEMENTARY FIGURES AND LEGENDS**


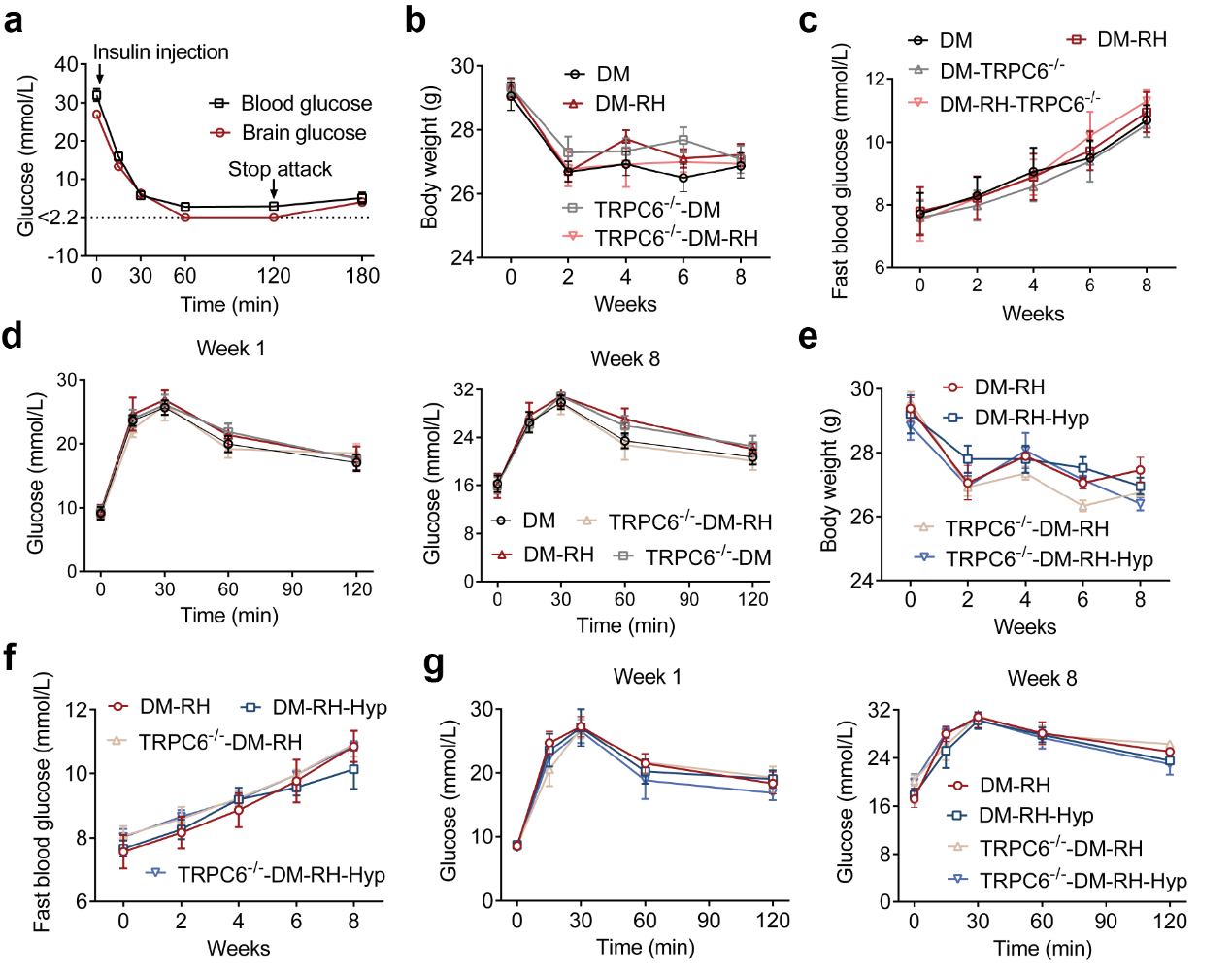


**Supplemental Fig. 1. The metabolic effects of RH and hyperforin treatment on diabetic mice. (a)** Changes of blood glucose and brain glucose after insulin injection in DM mice. A moderate hypoglycemia event induced by insulin (2.2-3.9 mmol/L) lasts for about 120 mins and stop by free access to food at the time of 120 mins. **(b and c)** Change of body weights (b) and fasting blood glucose (c) from week 1 (the begin of RH treatment) to week 8 (end of RH treatment). **(d)** The OGTT test preformed at the start (week 1, left) and end (week 8, right) of the RH treatment (n=6 mice). **(e and f)** Body weights (E) and fasting blood glucose (f) in hyperforin treatment mice (n=6 mice). **(g)** The OGTT test performed at the begin (week 1, left) and end (week 8, right) of hyperforin administration (n=6 mice). The data are expressed as the mean ± SEM. Statistical significance was assessed using a One-way ANOVA followed by Dunnett’s multiple comparisons test.


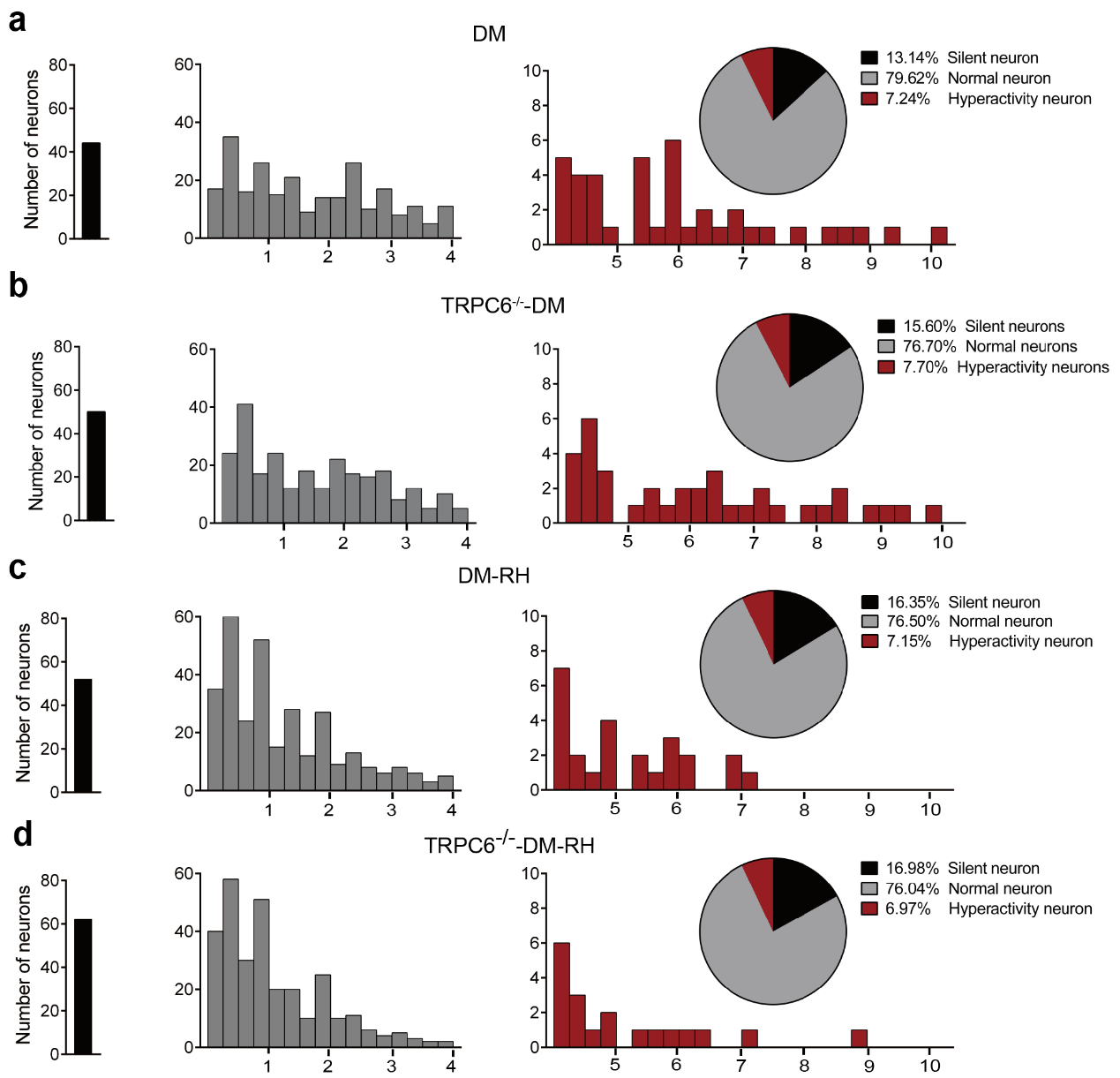


**Supplemental Fig. 2. The proportion of three type neurons in PFC is not significantly changed by RH or TPRC6 knockout in diabetic mice. (a-d)** Histograms showing the frequency distribution of spontaneous somatic Ca^2+^ transients of neurons, recorded by two-photon imaging, in the layer 2/3 of PFC in mice from the DM group (340 neurons from 8 mice, a), TRPC6^-/-^-DM group (393 neuons from 8 mice, b) and DM-RH group (362 neurons from 6 mice, c) and DM-RH-TRPC6^-/-^ group (380 neurons from 8 mice, d). Insets, Pie charts showing the relative proportion of silent, normal, and hyperactive neurons. Statistical significance was assessed by using a One-way ANOVA followed by Dunnett’s multiple comparisons test.


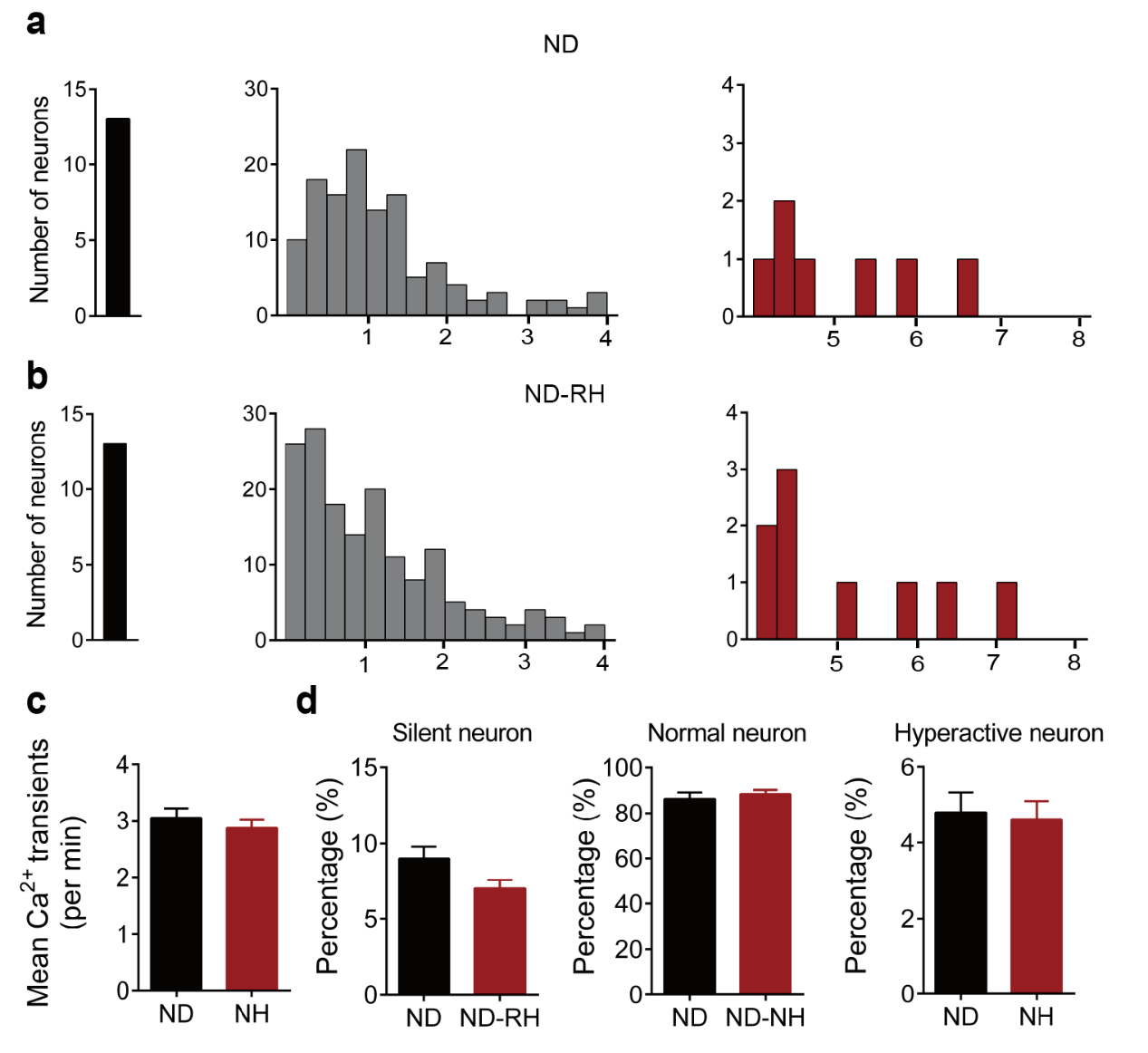


**Supplemental Fig. 3. RH has no effects on cortical neuronal activity in non-diabetic mice. (a and b)** Histograms showing the frequency distribution of spontaneous somatic Ca^2+^ transients of neurons in the layer 2/3 of PFC of mice from the ND group (n=145 neurons from 3 mice, a) and ND-RH group (n=182 neurons from 4 mice, b). **(c)** The mean of spontaneous somatic Ca^2+^ transients of neuron in layer 2/3 of PFC. **(d)** The percentage of silent, normal and hyperactive neurons in layer 2/3 of PFC**.** Statistical significance was assessed by using two-tailed Student’s t-test.

**·**


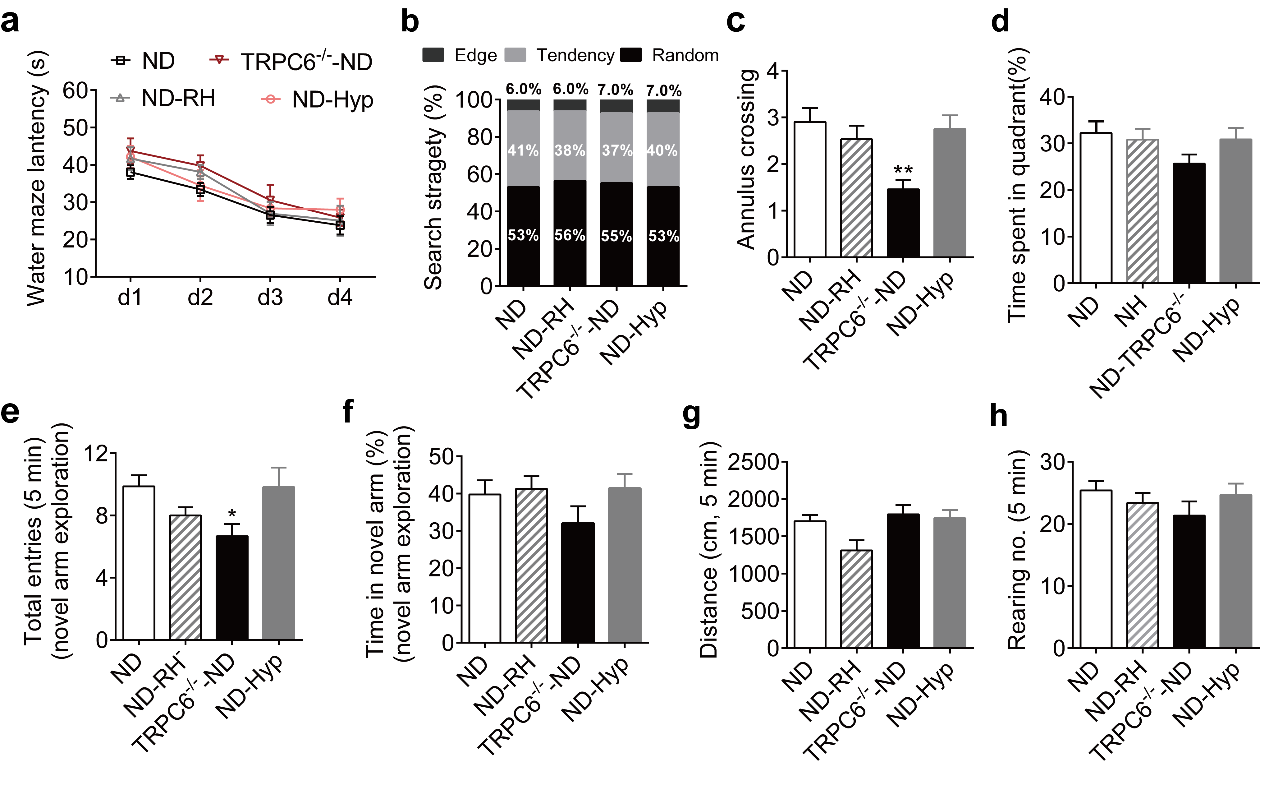


**Supplemental Fig. 4. Cognitive function in non-diabetic mice is not impaired by RH. (a and b)** Escape latency and searching strategy to find hidden platform during platform trials of the Morris water maze test in mice from ND, ND-RH, TRPC6^-/-^-ND and ND-Hyp group. **(c and d)** Number of annulus crossings (c) and time spent in the target quadrants (d) during the probe trial of the Morris water maze test. **(e and f)** Quantification of total entries (e) and percentage of time spent in the novel arm (f) during the novel arm exploration trials of the Y-maze test. **(g and h)** Distance traveled in the open field test and rearing number (h). (n=6-8 mice). The data are expressed as the mean ± SEM. Statistical significance was assessed by using two-tailed Student’s t-test. **p* < 0.05, ***p* < 0.01 compared with ND group.


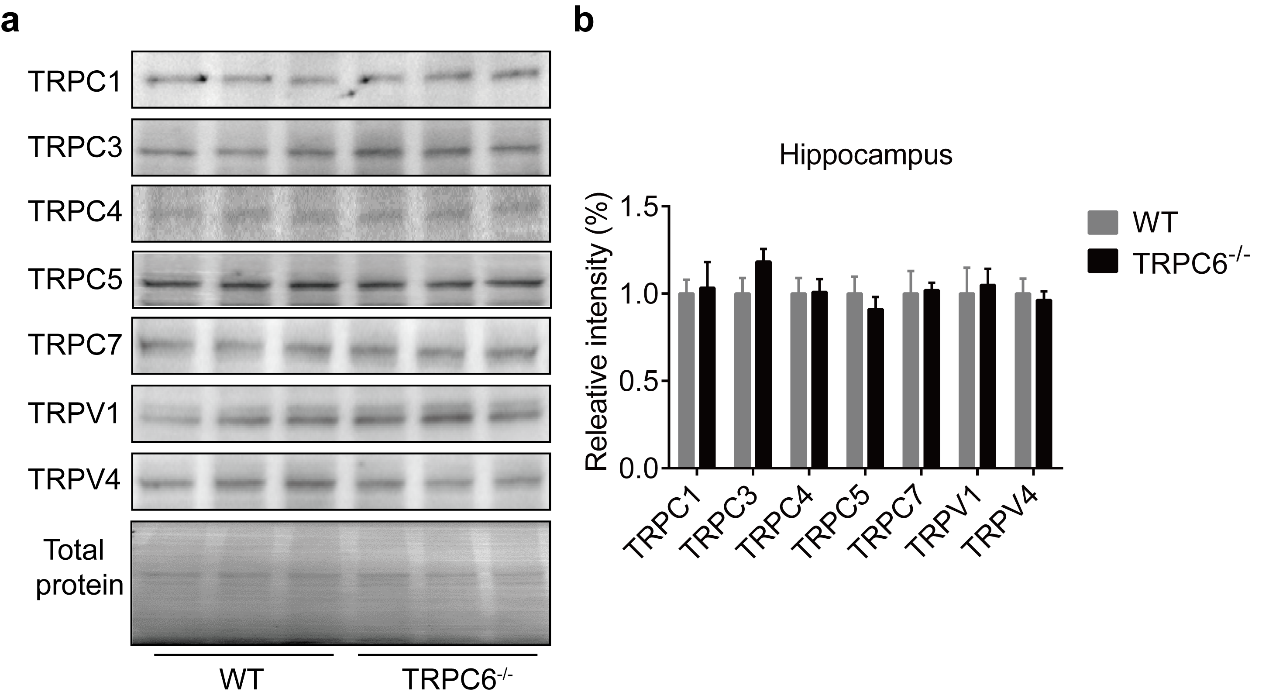


**Supplemental Fig. 5. TRP channels were not compensative up-regulation by TRPC6 knockout in hippocampus. (a)** Representative western blot images for TRPC1, TRPC3, TRPC4, TRPC5, TRPC7, TRPV1 and TRPV4 in hippocampal homogenates from WT and TRPC6^-/-^ mice. **(b)** Quantitative data of protein expression showed on the right (n=3 sample from 3 mice). Total protein was shown by Ponceau S solution staining. Statistical significance was assessed by using non-parametric tests.


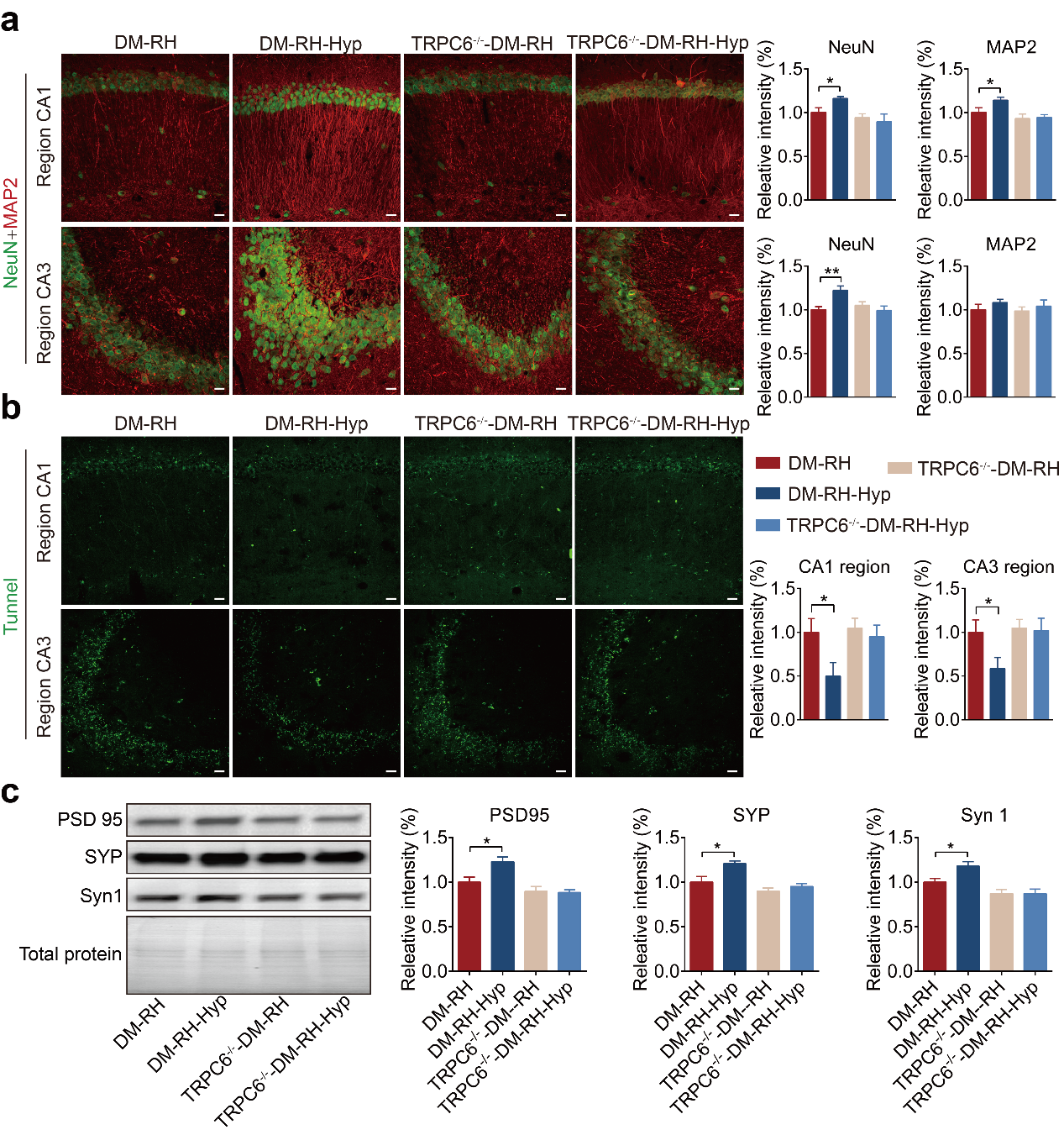


**Supplemental Fig. 6. Hyperforin treatment improves RH-caused neuronal loss in hippocampus in TRPC6 dependent manner. (a)** Representative image for neuronal loss detected by NeuN (neuron) and MAP2 (dendrite) immunostaining in CA1 region (*upper*) and CA3 region (*lower*) of hippocampus from indicated groups. The quantitative results showed on the right (n=6 samples from 6 mice). *Scale bar,* 50 um. (b) Representative image for neuronal apoptosis detected by TUNEL staining in CA1 and CA3 of hippocampus. Quantitative data was shown on the right (n=6 sample from 6 mice). *Scale bar,* 50 um. (C) Western blot for and quantification for synapse-associated proteins including PSD95, synaptophysin (SYP), Synapsin I (Syn I), and SNAP25 in hippocampal homogenates (n = 3 samples from 3 mice). **p* < 0.05, ***p* < 0.01. Statistical significance was assessed using a one-way ANOVA or Kruskal-Wallis test.


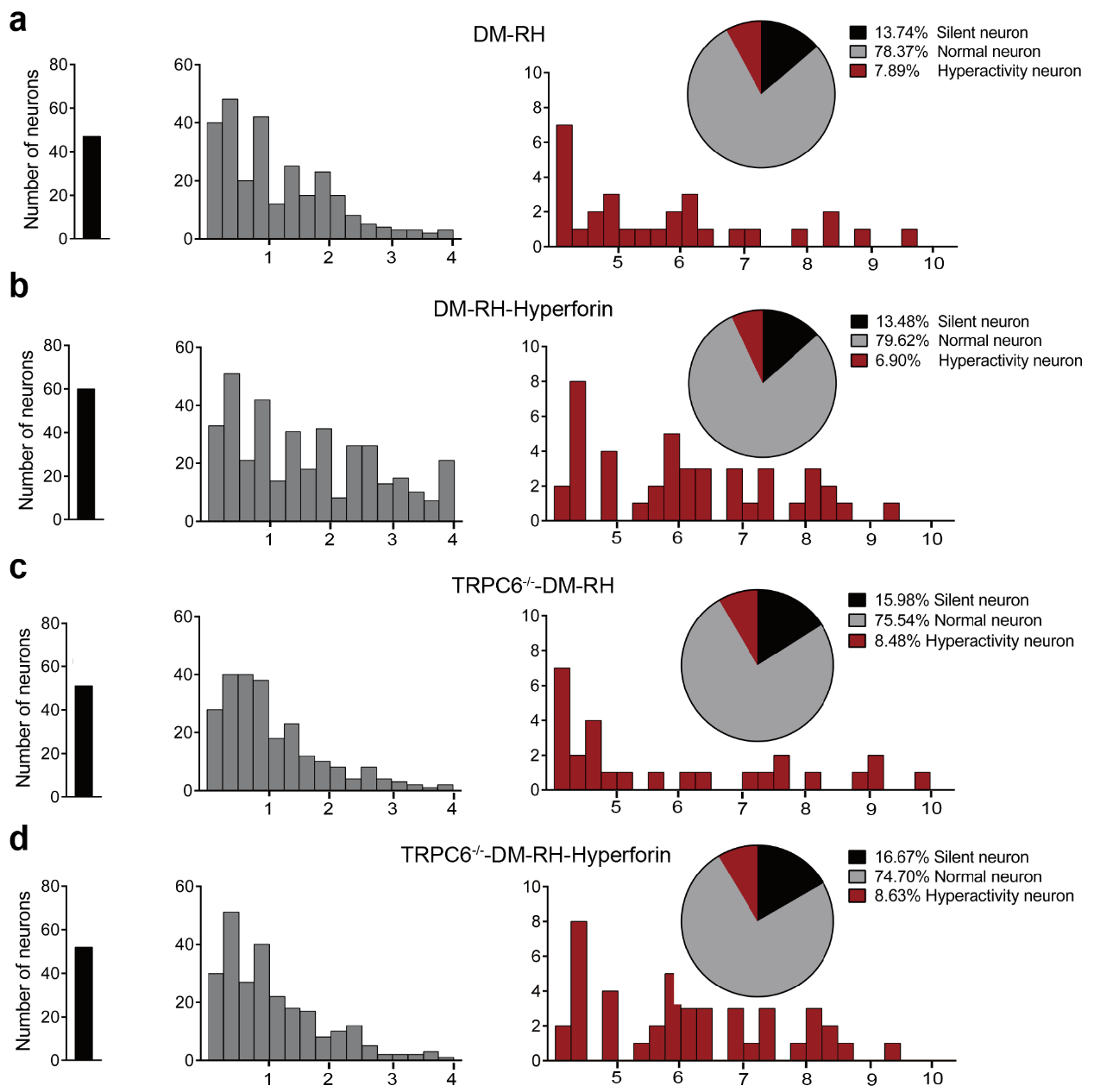


**Supplemental Fig. 7. The proportion of three type neurons in PFC is not significantly changed by hyperforin in diabetic mice. (a-d)** Histograms showing the frequency distribution of spontaneous somatic Ca^2+^ transients of neurons in the layer 2/3 of PFC of mice from the DM-RH group (n=344 neurons from 6 mice, a), DM-RH-Hyperfroin group (n=461 neurons from 9 mice, b) and TRPC6^-/-^-DM-RH group (n=319 neurons from 7 mice, c) and TRPC6^-/-^-DM-RH-Hyperforin group (417 neurons from 7 mice, d). Insets, Pie charts showing the relative proportion of silent, normal, and hyperactive neurons. Statistical significance was assessed by using two-tailed Student’s t-test.


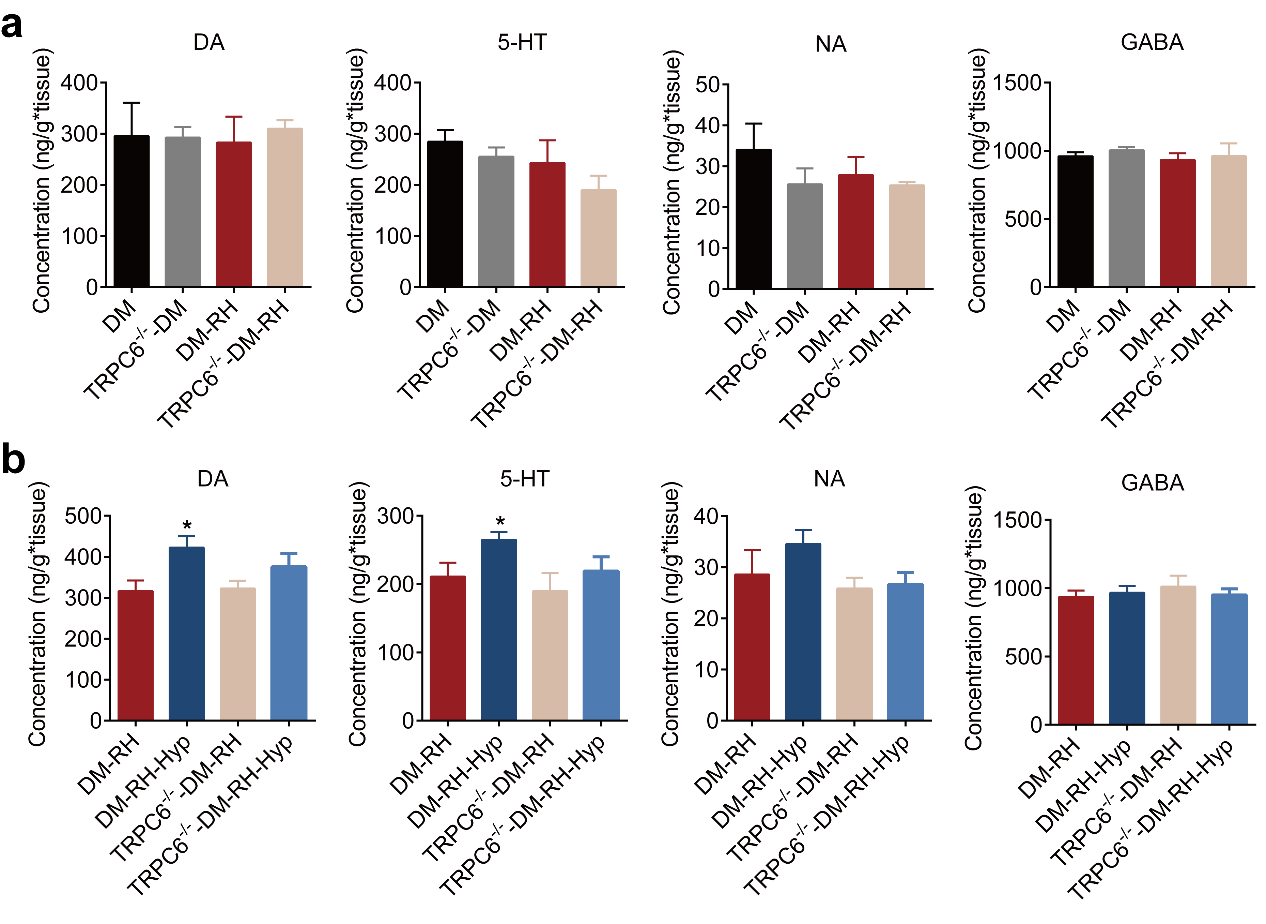


**Supplemental Fig. 8. Hyperforin increases the level of dopamine (DA) and 5-hydroxytryptamine (5-HT) in hippocampus in TRPC6 independent manner. (a and b)** The level of several neurotransmitter, including dopamine (DA), 5-hydroxytryptamine (5-HT), noradrenaline (NA) and gamma-aminobutyric acid (GABA), in hippocampal homogenates in mice from indicated groups (n= 6 sample from 6 mice). *p* < 0.05, ***p* < 0.01. Statistical significance was assessed using One-way ANOVA followed by Dunnett’s multiple comparisons test.


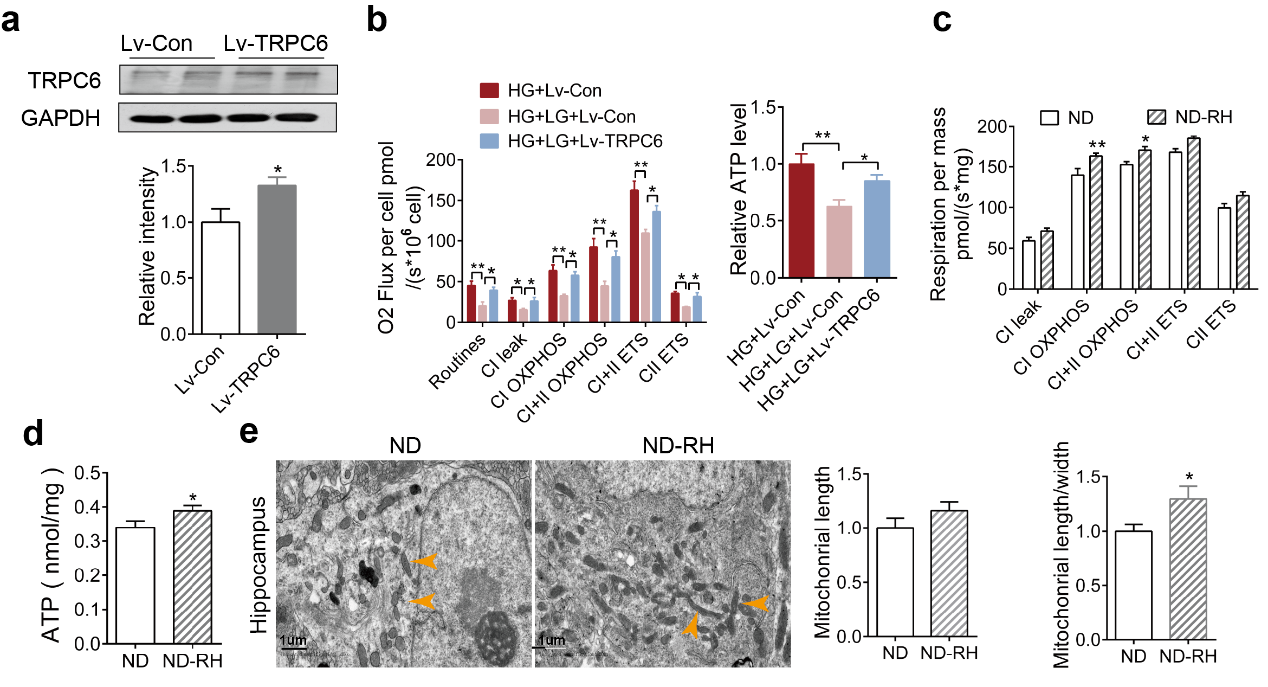


**Supplemental Fig. 9. The mitochondrial function in PC12 cells and morphology in the CA1 region of hippocampus from non-diabetic mice.** **(a)** TRPC6 protein expression in PC12 cells with or without TRPC6 over-expression, as determined by western blot analysis. Quantitative results showed on the right (n = 3). Lv-TRPC6, TRPC6 overexpression with lentivirus. **(b)** Summarized data for the oxygen consumption capacity of mitochondria measured by oxygraph-2k high-resolution respirometry and ATP content in PC12 cells (n = 3). **(c and d)** The oxygen consumption capacity of mitochondria (c) and ATP content (d) in hippocampus of mice from ND and ND-RH group (n = 4 tissues form 4 mice). **(e)** Transmission electron microscope (TEM) images of mitochondria (indicated by arrows) in the CA1 region of hippocampus of mice from ND and ND-RH group. Quantitative results of mitochondrial length and ratio of length to width from electron microscope images are showed on the right (n=80-120 mitochondria from 3 mice). Scale bar,1um. **p* < 0.05, ***p* < 0.01. Statistical significance was assessed using a Two-way ANOVA or One-way ANOVA followed by Dunnett’s multiple comparisons test or two-tailed Student’s t-test.


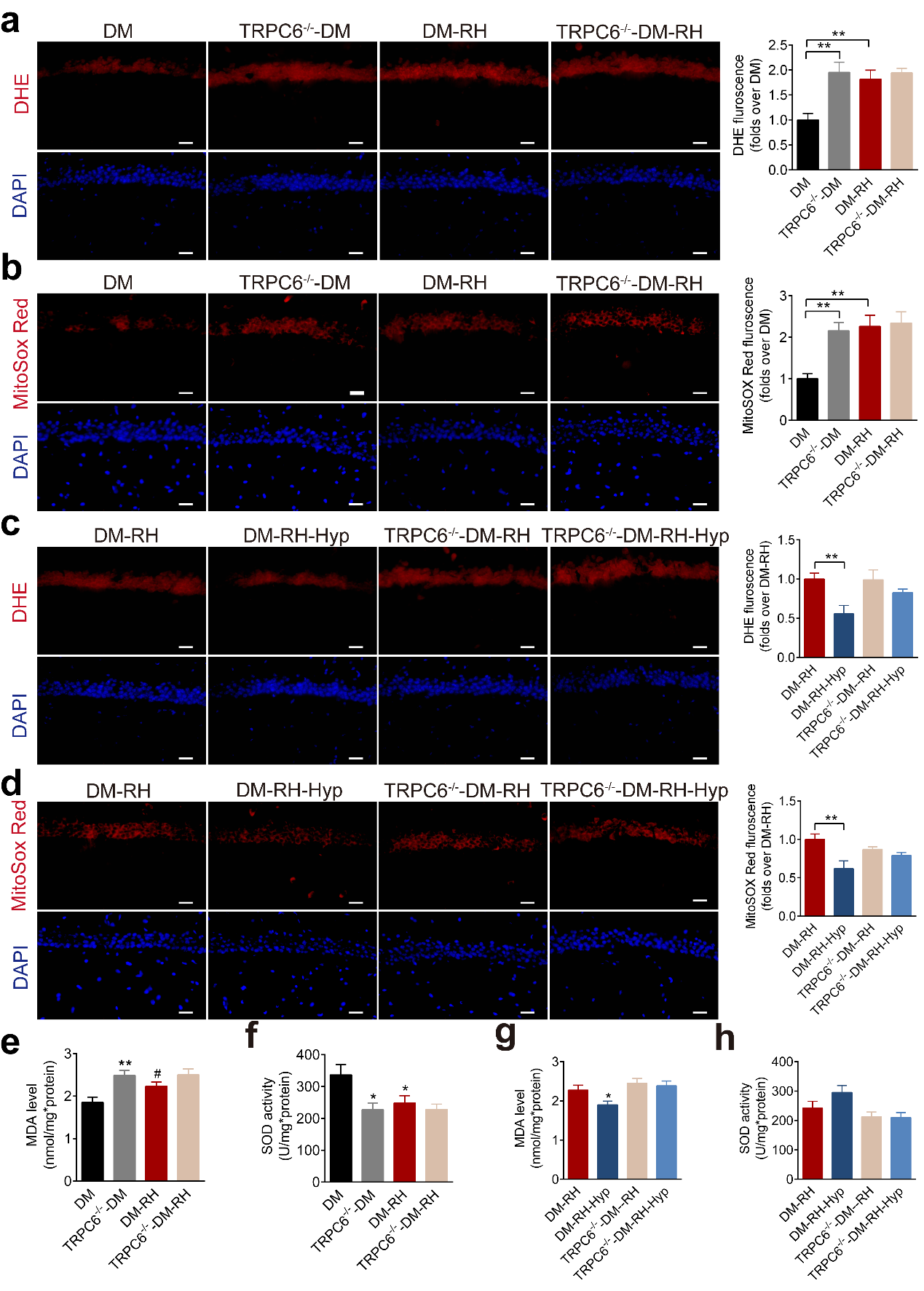


**Supplemental Fig. 10. Hyperforin treatment alleviates RH-induced oxidative stress in hippocampus.** **(a-d)** Cytosolic superoxide (a, c) measured by dihydroethidium (DHE) and mitochondrial superoxide (b, d) measured by MitoSOX Red in hippocampus from indicated groups. Nucleus was stained by DAPI. Quantitative data was shown on the right (n=6 samples from 6 mice). **(e and g)** Malondialdehyde (MDA) level in hippocampal homogenates (n=4 samples 4 mice). **(f and h)** Superoxide dismutase (SOD) activity in hippocampus (n=4 samples 4 mice). **p* < 0.05, ***p* < 0.01. Statistical significance was assessed using One-way ANOVA followed by Dunnett’s multiple comparisons test.


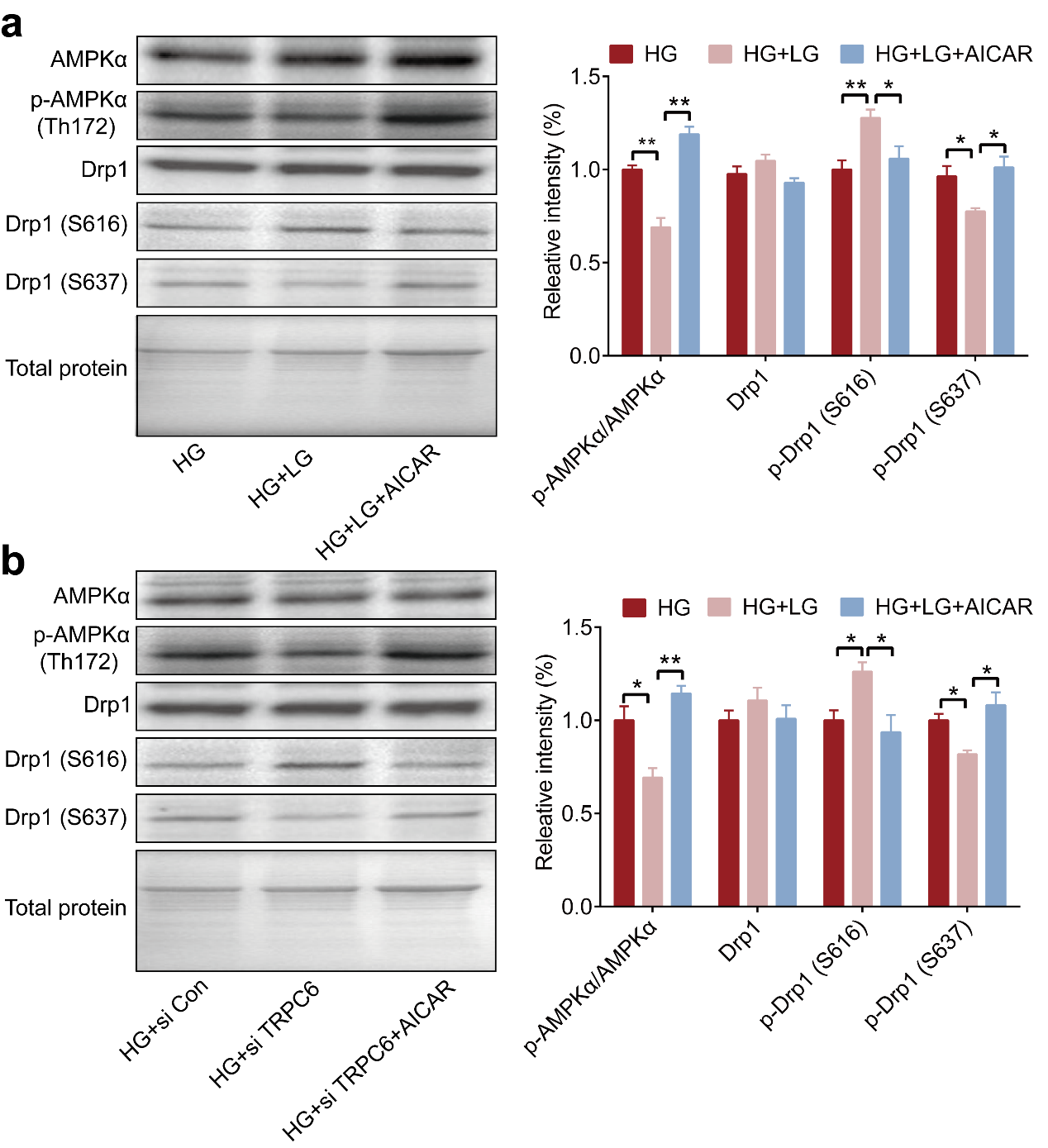


**Supplemental Fig. 11. Activation of AMPK with AICAR blocks the change of Drp1 phosphorylation caused by repeated LG stimulation or TRPC6 down-regulation. (a)** Representative western blots showed the expression of AMPKα, p-AMPKα, Drp1, p-Drp1 (S616), p-Drp1 (S637) in PC12 cells treated with repeated LG and/or AICAR (1 mM). Quantitative data were shown on the right (n=3). **(b)** Representative western blots showed the expression of AMPKα, p-AMPKα, Drp1, p-Drp1 (S616), p-Drp1 (S637) in PC12 cells with TRPC6 down-regulation and/or AICAR (1 mM). Quantitative data was shown on the right (n=3). **p* < 0.05, ***p* < 0.01, ****p* < 0.01. Statistical significance was assessed using Kruskal-Wallis test followed by Dunn’s multiple comparisons test.


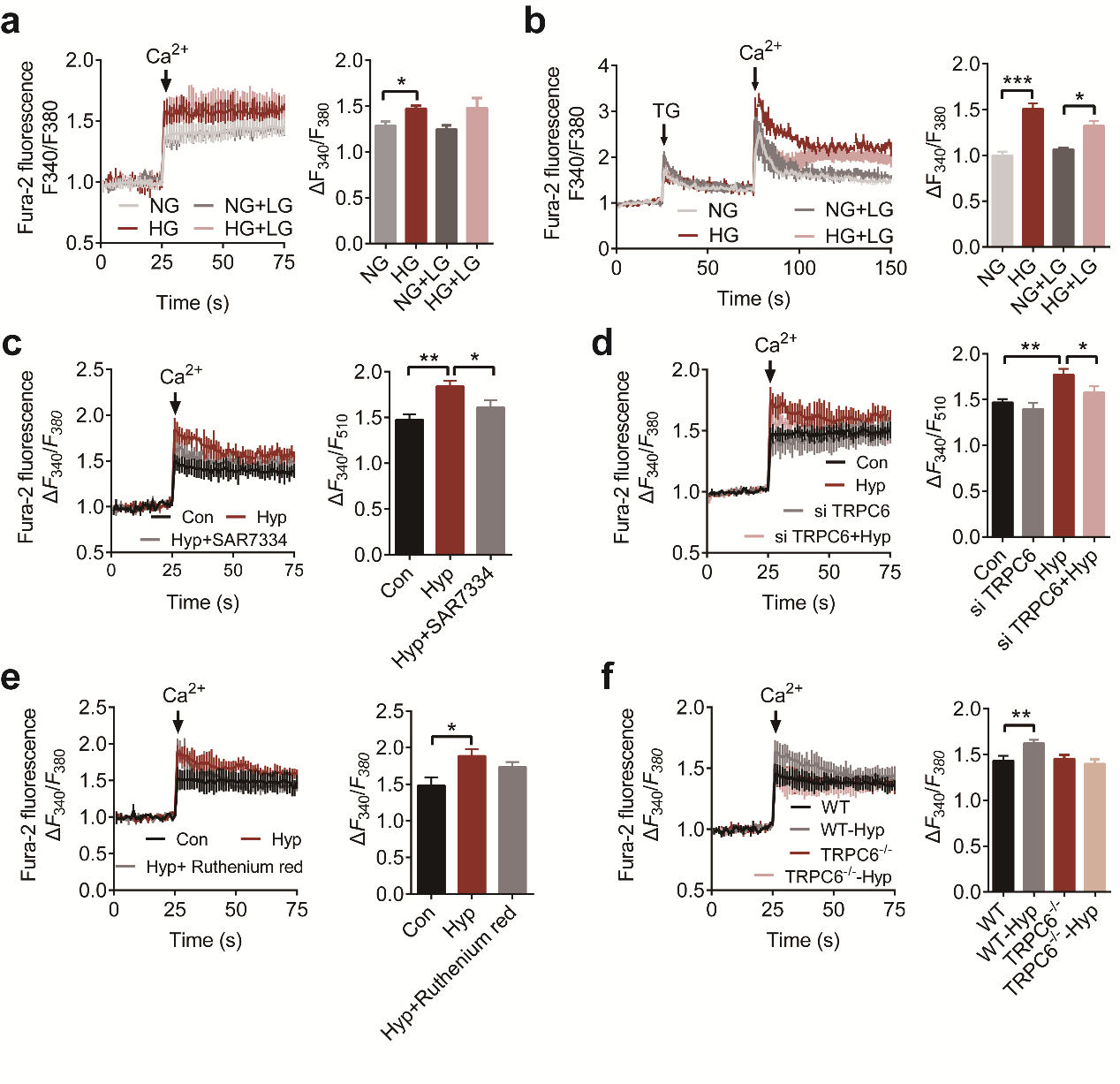


**Supplemental Fig. 12. Hyperforin-induced cytosolic Ca^2+^ influx was mediated by TRPC6 activation. (a and b)** HG culturing induced persistent and bulk cytosolic Ca^2+^ elevation. Quantitative data were shown on the right (n=3).TG, thapsigargin. **(c and d)** SAR7334 (100 nM) or TPPC6 down-regulation with siRNA significantly blocked hyperforin-induced cytosolic Ca^2+^ influx in PC12 cells. Quantitative data were shown on the right (n=3). SAR7334, an inhibitor for TRPC6; Hyp, hyperforin (10 uM); Ca^2+^, Cacl_2_, 1mM. **(e)** Hyperforin-induced Ca^2+^ influx was completely absent in primary hippocampal neurons from TRPC6^-/-^ newborn mice**.** Quantitative data were shown on the right (n=3).  **(f)** Ruthenium red did not affect hyperforin-induced cytosolic Ca^2+^ influx in PC12 cells. Quantitative data were shown on the right (n=3). **p* < 0.05, ***p* < 0.01, ****p* < 0.001, Statistical significance was assessed using Kruskal-Wallis test followed by Dunn’s multiple comparisons test.

**Supplemental Table 1. Basic physiological parameters of participants.**

| Characteristics | DM-RH patients  (n=16) | DM Patients  (n=22) | Normal Controls  (n=18) |
| --- | --- | --- | --- |
| Age (Years) | 63.2±7.43 | 63.4±6.5 | 62.0±143 |
| Gender(male/female) | 8/8 | 10/12 | 9/9 |
| BMI（kg/m^2^） | 24.0±3.6 | 25.0±2.3 | 24.2±4.7 |
| FPG（mmol/L） | 6.69±2.39 | 8.39±2.98**^#^** | 4.87±0.46 |
| FINS（uU/ml） | 14.3±9.0 | 17.8±4.2 | 16.2±3.6 |
| HbA1c（%） | 8.9±2.0* | 8.3±1.6**^#^** | 5.8±0.4 |
| SBP（mmHg） | 134±12 | 135±17 | 125±18 |
| DBP（mmHg） | 77±9 | 77±10 | 76±10 |
| TC（mmol/L） | 4.56±1.17 | 5.29±1.00 | 5.39±0.92 |
| TG（mmol/L） | 1.40±0.60 | 1.38±0.53 | 1.29±0.64 |
| HDL-c（mmol/L） | 1.21±0.18 | 1.23±0.23 | 1.36±0.04 |
| LDL-c（mmol/L） | 2.69±1.06 | 3.10±0.84 | 2.48±1.19 |

The results showed as Mean ± SD. *P* values are obtained by Kruskal-Wallis test. BMI, body mass index; FPG, fast plasma glucose; **p* < 0.05 compared with T2DM patients. ^#^*p* < 0.05 compared with normal controls.

**Supplemental Table 2. Cognitive function scales.**

| DM-RH patients DM patients Normal Controls  (n =16) (n =22） (n =18） |
| --- |
| MMSE 26.81±1.42 27.62±1.64**^#^** 28.9±1.1  MES-T 82.25±10.27***** 90.1+7.55 92.5±6.65  MES-M 34.56±9.43 40.95±6.61 43±6.36  MES-E 47.69±2.47 48.86±1.93**^#^** 49.5±1.08  RAVL 22.63±4.18 25.63+5.64 30.2±7.11  RVL 3.19±2.37*****  5.75±3.4**^#^** 7.33±2.98  LDFR 4.75±1.69 6.35±2.64 7.78+2.39 |

MMSE, Mini-mental State Examination; MES-T, Memory and Execution screening-Total; MES-M, Memory and Execution screening-Memory; MES-E, Memory and Execution screening-Execution; RAVL, Rey auditory verbal learning; RVL, Recognitive verbal learning; LDFR, Long delay free recall. The results showed as Mean ± SD. *P* values are obtained by Kruskal-Wallis test. **p* < 0.05 compared with T2DM patients. ^#^*p* < 0.05 compared with normal controls.
